# FT-ICR/MS and GC-EI/MS Metabolomics Networking Unravels Global Potato Sprout's Responses to *Rhizoctonia solani* Infection

**DOI:** 10.1371/journal.pone.0042576

**Published:** 2012-08-03

**Authors:** Konstantinos A. Aliferis, Suha Jabaji

**Affiliations:** Department of Plant Science, McGill University, Sainte-Anne-de-Bellevue, Quebec, Canada; Soonchunhyang University, Republic of Korea

## Abstract

The complexity of plant-pathogen interactions makes their dissection a challenging task for metabolomics studies. Here we are reporting on an integrated metabolomics networking approach combining gas chromatography/mass spectrometry (GC/MS) with Fourier transform ion cyclotron resonance/mass spectrometry (FT-ICR/MS) and bioinformatics analyses for the study of interactions in the potato sprout-*Rhizoctonia solani* pathosystem and the fluctuations in the global metabolome of sprouts. The developed bioanalytical and bioinformatics protocols provided a snapshot of the sprout's global metabolic network and its perturbations as a result of pathogen invasion. Mevalonic acid and deoxy-xylulose pathways were substantially up-regulated leading to the biosynthesis of sesquiterpene alkaloids such as the phytoalexins phytuberin, rishitin, and solavetivone, and steroidal alkaloids having solasodine and solanidine as their common aglycons. Additionally, the perturbation of the sprout's metabolism was depicted in fluctuations of the content of their amino acids pool and that of carboxylic and fatty acids. Components of the systemic acquired resistance (SAR) and hypersensitive reaction (HR) such as azelaic and oxalic acids were detected in increased levels in infected sprouts and strategies of the pathogen to overcome plant defense were proposed. Our metabolic approach has not only greatly expanded the multitude of metabolites previously reported in potato in response to pathogen invasion, but also enabled the identification of bioactive plant-derived metabolites providing valuable information that could be exploited in biotechnology, biomarker-assisted plant breeding, and crop protection for the development of new crop protection agents.

## Introduction

It is estimated that the metabolomes of higher plants in total are composed of more than 100,000 primary and secondary metabolites out of which approximately 10% have been identified to date. Many of these metabolites are key components of the plant's defense [1], and could constitute a rich source of bioactivity of high potential for various applications in biotechnology, biomarker-assisted plant breeding, and crop protection. The quantitative and qualitative composition of plant metabolomes reflects their genome, physiological status, and responses to biotic and abiotic stimuli, thus serving as the link between genotypes and phenotypes. However, their complexity makes their comprehensive monitoring a challenging task which requires the development and hyphenation of powerful and high-throughput bioanalytical protocols. Currently, there is no single bioanalytical protocol and analyzer capable of monitoring the complete range of metabolites that exist in plant tissues, and this is the main reason that metabolomics has not yet been fully exploited especially in the study of plant-pathogen pathosystems.

Metabolomics is a recently developed tool of systems biology which has enriched our knowledge on the regulation of metabolic networks [2], [3], [4], [5], [6]. One of its novel features is the quest of system-wide mapping of metabolites which has been facilitated by advances in high-throughput analytics. Until now, a great deal of effort has been made towards the standardization of metabolomics [7] in order to provide information on the functional linkages between genome, transcriptome, proteome, and phenome. However, the robust biological interpretation of the vast amount of information obtained by metabolomics remains a challenge.

Plant-pathogen interactions are interesting in terms of metabolite richness and metabolism regulation and can serve as an ideal model for the development and standardization of high-throughput metabolomics. To date, a small number of metabolomics studies on plant-pathogen interactions have been published [8], [9], [10], [11], [12] and the research on the topic has been recently reviewed [13], [14], [15].

Potato (*Solanum tuberosum* L.) is among the most important food crops consumed worldwide (http://faostat.fao.org/site/339/default.aspx). In addition to nutrients, the plant synthesizes various bioactive secondary metabolites among which alkaloids are the most extensively studied with respect to their bioactivity, toxicology, and role in plant's physiology [16]. Attack by microorganisms trigger the plant's defense mechanism leading to biosynthesis of secondary metabolites derived from the isoprenoid, phenylpropanoid, alkaloid or fatty acid/polyketide pathways [1]. There has been several studies on metabolite profiling in potato in order to annotate gene function through comparing metabolite composition of transgenic potato with that of wild true plants [17], [18], fingerprinting of potato genotypes [19], studying developmental processes and wound induced metabolism [20], [21], [22], and analysis of targeted metabolite/flux on tuber development [23], [24]. However to date, there exist no metabolomic studies on the effects of pathogens on the global metabolic network of potato.

*Rhizoctonia* diseases of potato occur wherever potatoes are grown, and are caused by the fungus *Rhizoctonia solani* anastomosis group 3 (AG-3). They are found on all subterranean parts of the plant causing black scurf on tubers and canker on underground stems and stolons [25]. Damage to infected plants is materialized as poor stands, malformed tubers and considerable reduction of the marketable yield [26]. Stem and stolon canker is the most damaging phase of the disease as it occurs underground prior to emergence and often goes unnoticed. Early in disease development infection pegs occur followed by inter- and intracellular growth that is associated with secretion of extracellular enzymes and development of dark brown necrotic lesions on the lower parts of the stems and stolons [27]. There have been few studies reporting on the induction of plant defence responses as a result of *R. solani* infection [27], [28], [29]. Recently, Lehtonen et al. [30] reported on the differential expression of well-characterized defense genes during infection of subterranean potato sprouts with a virulent strain of *R. solani*, leading to systemic induction of resistance in sprouts upon infection. However, knowledge on their end-products implicated in plant defense responses is non-existent.

Since information on metabolic profiling of potato sprouts under the influence of *R. solani* is lacking, the goal of this study was to characterize changes in the metabolome of sprouts in relation to disease and present global knowledge on a metabolomics networking approach by integrating Fourier transform ion cyclotron resonance/mass spectrometry (FT-ICR/MS) analyses with gas chromatography/mass spectrometry (GC/MS), in which the topologies and regulatory activities of the metabolic networks are mapped during potato sprout's interaction with *R. solani*.

## Results

### Statistical analyses reveal distinct metabolic profiles between control and *Rhizoctonia solani*-infected sprouts and corresponding biomarkers

**Figure 1:**
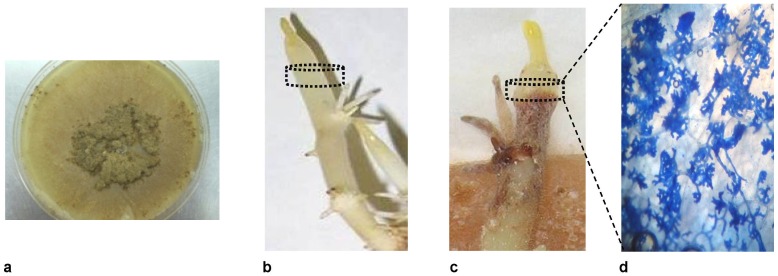
*Rhizoctonia solani* AG3 culture used as the inoculum (a), healthy and non-infected sprout (b), infected potato sprout 72 h after inoculation with the pathogen (c). The portion of the sprout between the dashed lines was subjected to metabolomics analyses. In infected sprouts, samples included a small portion of the necrotic lesion. A necrotic region showing hyphae and infection cushions magnified at 600× (d).

**Figure 2:**
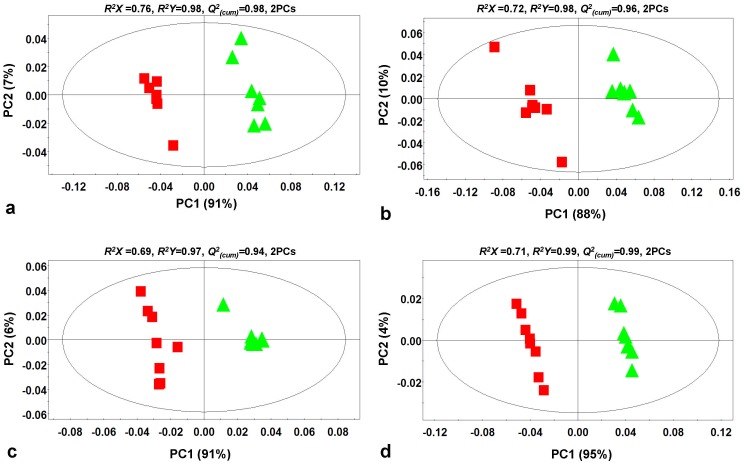
Partial least squares-discriminant analyses (PLS-DA) PC1/PC2 score plots of FT-ICR/MS metabolic profiles recorded in positive (a) and negative (b) modes, GC/MS metabolic profiles (c), and combined FT-ICR/MS and GC/MS metabolic profiles (d) of healthy (▴) and *Rhizoctonia solani* infected (▪) potato sprouts. The ellipse represents the Hotelling T^2^ with 95% confidence interval. Eight (8) biological replications were performed per treatment [*Q^2^_(cum)_*; cumulative fraction of the total variation of the *X*'s that can be predicted by the extracted components, *R^2^X* and *R^2^Y*; the fraction of the sum of squares of all *X*'s and *Y*'s explained by the current component, respectively].

Fluctuations in the potato sprout's metabolome in response to *R. solani* infection (Fig. 1) were recorded by integrating FT-ICR/MS and GC/MS (Figure S1) using bioinformatics software and metabolite species-specific databases. Such approach provides solid evidence that the use of more than one analyzer expands metabolite coverage and strengthens identification confidence. In total, 270 metabolites belonging to various chemical groups were putatively or tentatively identified and ions were assigned to unique chemical formulae. In total, four data matrices were constructed from the pre-processed MS data [FT-ICR/MS-positive electrospray ionization (ESI^+^)-161 rows×16 columns, FT-ICR/MS-negative electrospray ionization (ESI^−^)-121 rows×16 columns, GC/MS-162 rows×16 columns, and combined FT-ICR/MS-GC/MS-444 rows×16 columns]. The experimental protocol proved to be valid as indicated by the high values of explained variation (*R^2^X*) and predictive ability [*Q^2^_(cum)_*] (*P*<0.05) performing multivariate analyses (Fig. 2, Figure S2) and the tight clustering performing hierarchical cluster analyses (HCA) (Figure S3).

**Figure 3:**
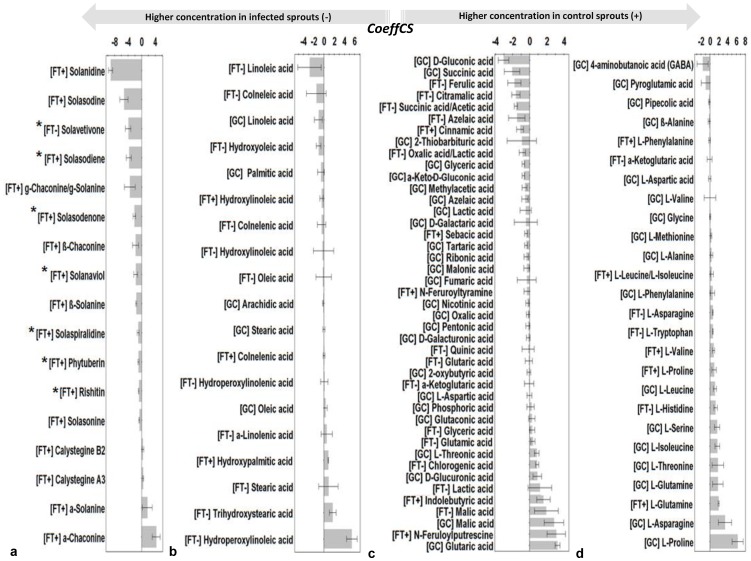
Partial least squares (PLS) coefficient plots for the combined FT-ICR/MS (positive and negative electrospray modes) and GC/MS data matrix with values of scaled and centered PLS regression coefficients (CoeffCS) for selected identified metabolites. Influential metabolites for the observed separation between the metabolomes of control and infected *Solanum tuberosum* sprouts by *Rhizoctonia solani* AG3 72 h post-infection are displayed with Jack-knifed confidence intervals (*P*<0.05) belonging to alkaloids (a), lipid acids and hydroperoxides (b), carboxylic acids and various metabolites (c), and amino acids (d). Metabolites marked with asterisk (*) denote metabolites detected exclusively in the infected spouts. Negative values of CoeffCS denote metabolites with higher concentration in infected sprouts whereas positive values those with higher concentration in non-infected sprouts.

Application of principal components analysis (PCA) revealed tight groups with no outliers (*P*<0.05) (Figure S2). Metabolites showing substantial fluctuation as well as *de novo* synthesized were designated as biomarkers of plant-pathogen interactions (Fig. 3 Table S1). For the purpose of this study, the term “*de novo* synthesized” in infected sprouts refers to metabolites not detected in healthy sprouts, without excluding the possibility of their presence in concentrations below the detection limits of the employed analyzer. The selection of biomarkers was based on partial least squares-discriminant analysis (PLS-DA) that showed an excellent discrimination between control and infected sprouts [*Q^2^_(cum)_* ranged between 0.94 and 0.99; Fig. 2] and PLS-DA regression coefficients (*P*<0.05), confirming the robustness and reliability of the developed model. Additionally, similar fluctuation in the metabolites composing the metabolic profiles recorded by FT-ICR/MS and GC/MS (Fig. 3) further supports the validity of metabolite identification. In agreement with PLS-DA, 2D-HCA revealed a strong clustering of the metabolomes of healthy and infected sprouts indicating distinctive differences in response to pathogen attack (Figure S3).

### *Rhizoctonia solani* infection activates biosynthetic pathways that lead to biosynthesis of alkaloids

**Figure 4:**
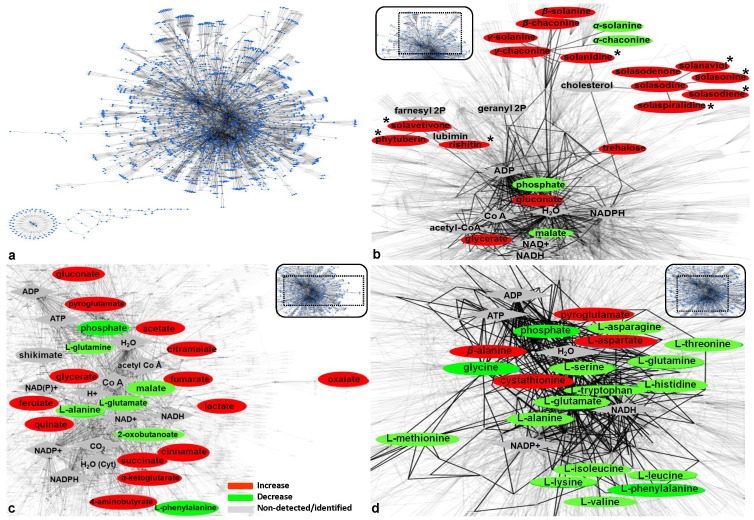
The *Solanum tuberosum* metabolome (A) visualized using the software Cytoscape (v.2.7.0.) and the reconstructed and curated PotatoCyc database. Changes in the sub-networks of sprouts' alkaloids (B), carboxylic (C), and amino acids (D) 72 h after infection by *Rhizoctonia solani* AG3 are displayed. All possible pathways between the detected metabolites are highlighted. Metabolite fluctuations are coded using a color code based on the means of scaled and centered PLS regression coefficients (CoeffCS) from eight replications. With asterisk (*) *de novo* produced metabolites are marked. Nodes represent metabolites, enzymes, nucleotides, CO_2_, H_2_O or reactions, while edges represent the connections between them.

**Figure 5:**
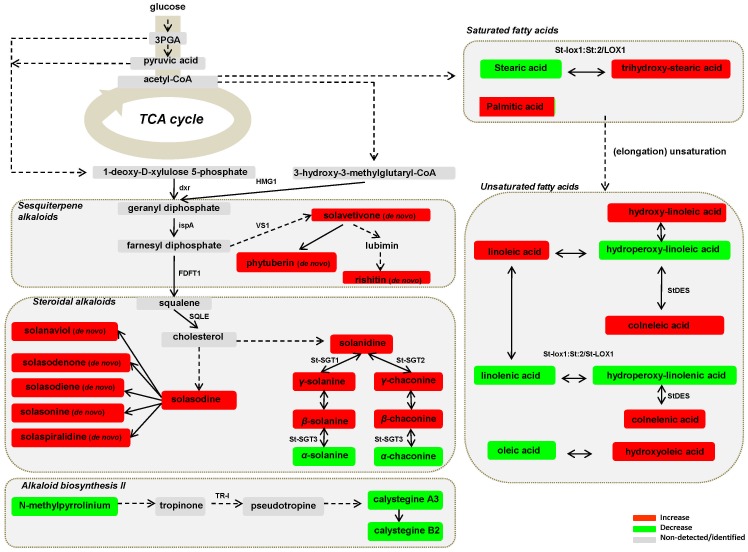
Fluctuations in *Solanum tuberosum* sprout metabolic pathways leading to the biosynthesis of sesquiterpene, steroidal, and nortropane alkaloids, saturated and unsaturated fatty acids and selected fatty acid oxidation products, 72 h after infection by *Rhizoctonia solani* AG3. Metabolite fluctuations are coded using a color code based on the means of scaled and centered PLS regression coefficients (CoeffCS) from eight replications. Dashed lines symbolize multistep or not fully elucidated reactions and solid lines one step reactions [3PGA; 3-phosphoglycerate, dxr; 1-deoxy-D-xylulose-5-phosphate reductoisomerase, FDFT1; farnesyl-diphosphate farnesyltransferase, ispA; farnesyl diphosphate synthase, HMG1; 3-hydroxy-3-methylglutaryl-CoA reductase, LOX; lipoxygenases, St-DES; 9-divinyl ether synthase, St-SGT1; galactose galactosyltransferase; St-SGT2; glucose glucotransferase, St-SGT3; rhamnosyltransferase, SQLE; squalene monooxygenase, TR-I; tropinone reductase I, VS1; vetispiradiene synthase].

In the main branch of the steroidal alkaloid biosynthetic pathway, a substantial increase (53.86%) in the common aglycon solanidine, the most abundant metabolite (30.59%) in FT-ICR/MS (ESI^+^) metabolite profiles of infected sprouts, was observed (Figs. 3a, 4b, 5 and Table S1). Concomitantly, the relative peak intensities of *β*- and *γ*-, chaconine and solanine significantly increased with a simultaneous decrease of their *α-* forms (*P*<0.05).

Intriguingly, *R. solani* infection resulted in the *de novo* synthesis of several steroidal alkaloids (Figs. 3a, 4b, 5 and Table S1) that have solasodine as their common aglycon such as solasodenone, solanaviol, solasodiene, solasonine, and solaspiralidine. Their relative peak intensity ranged between 0.06 and 1.17% in FT-ICR/MS (ESI^+^) metabolite profiles (Table S1). Also, an 88.81% increase of solasodine which represents 3.89% of the relative peak intensity of infected sprouts in FT-ICR/MS (ESI^+^) metabolite profiles was observed.

Complementary to the increased biosynthesis of terpenoid glycoalkaloids, the activation of mevalonic acid and deoxy-xylulose pathways towards the *de novo* biosynthesis of the following potato sesquiterpenoid phytoalexins: phytuberin, rishitin, and solavetivone at lesion sites, when sprouts are challenged by *R. solani*, was detected as another major sprout defense response. Their relative peak intensities ranged between 0.16 and 1.52% (Figs. 3a, 4b, 5 and Table S1). On the other hand, the levels of the bioactive nortropane alkaloids calystegines A3 and B2 levels decreased after infection (Figs. 3a, 5 and Table S1).

### The fatty acid and fatty acid hydroperoxide content of *Rhizoctonia solani*-infected sprouts significantly differs compared to that of control sprouts indicating their involvement in pathogenesis

Upon fungal challenge, the relative content of infected sprouts in fatty acids (FAs) and their corresponding oxidized forms (oxylipins) was considerably altered (Figs. 3b, 5 and Table S1). Unsaturated FAs were more affected compared to saturated ones, whose content slightly decreased after infection. Linoleic acid exhibited 44.34% and 26.32% increase in FT-ICR/MS (ESI^−^) and GC/MS metabolite profiles, respectively. However, the presence of linoleic acid in fungal hyphae (Table S2) may partially explain such increase. Notably, was the increase in the relative peak intensities of the fungitoxic colneleic (84.79%) and colnelenic (20.68%) acids, two major potato divinyl ether FAs, in infected sprouts. Increased concentrations of the oxidized saturated FAs forms with simultaneous decrease of the unsaturated FAs forms were also recorded (Figs. 3b, 5). However, healthy sprouts had higher content of hydroperoxylinoleic acid compared to the infected.

### The content of the majority of carboxylic acids increases in sprouts under the influence of *Rhizoctonia solani*

**Figure 6:**
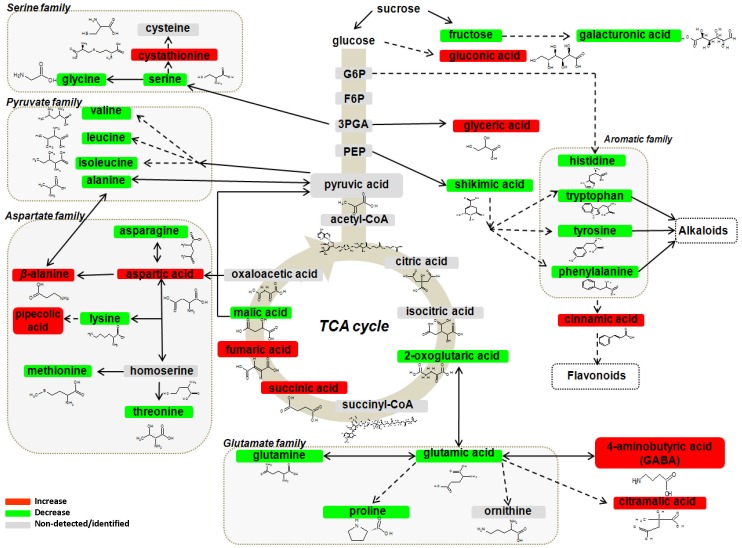
Changes in the protein and non-protein amino acid pools of infected *Solanum tuberosum* sprouts 72 h after infection with *Rhizoctonia solani* AG3. Fluctuations in metabolite relative concentration are coded using a color code based on the means of scaled and centered PLS regression coefficients (CoeffCS) from eight replications. Dashed lines symbolize multistep or not fully elucidated reactions and solid lines one step reactions [3PGA; 3-phosphoglycerate, F6P; fructose-6-phosphate, G6P; glucose-6-phosphate, PEP; phosphoenolpyruvate].

The majority of the identified carboxylic acids increased in infected sprouts (Figs. 3c, 4c, 6 and Table S1). Notably, were the increases in citramalic (92.75%) and succinic acids (65.36%) in GC/MS metabolic profiles. However, the increase in succinic acid could be partially attributed to fungal derived primary metabolites (Table S2). Infected sprouts had also increased content in azelaic, oxalic, gluconic, and *α*-keto-*d*-gluconic acids (Fig. 3). In contrast, decreased concentrations of glucuronic and galacturonic acids, which are uronic derivatives of glucose and galactose respectively, and building blocks of cell wall polysaccharides, were observed following infection (Table S1). Also, notable decreases in the levels of malic (71.13%) and indolebutyric (62.75%) acids were observed in infected sprouts.

### *Rhizoctonia solani* infection causes a decrease of the protein amino acid pool of sprouts with concomitant increase of that of non-protein amino acids

The content of protein amino acid pool was significantly decreased in infected compared to the non-infected sprouts with the exception of pyroglutamic acid (Figs. 3d, 4d, 6 and Table S1). Although several amino acids were detected as fungal components (Table S2), the observation does not make the interpretation of results problematic since a general decrease was recorded for the vast majority of protein amino acids. The highest decline was observed for L-histidine (89.74%), L-proline [79.17% in FT-ICR/MS ESI^+^ and 42.87% in GC/MS profiles], and L-valine [68.56% FT-ICR/MS (ESI^+^) profiles]. The cyclic amino acid pyroglutamic acid whose biosynthesis is catalyzed by aminoacyltransferase (E.C. 2.3.2.-) was the only protein amino acid which increased following infection.

Similarly, GC/MS profiles of infected sprouts showed a considerable increase in the non-protein amino acids *β*-alanine (20.05%), 4-aminobutyric acid (GABA) (20.87%), and pipecolic acid (33.18%) compared to those of non-infected sprouts.

### Changes in the content of carbohydrates in sprouts following *Rhizoctonia solani* invasion is indicative of a general remobilization of sugars

A limitation performing direct infusion FT-ICR/MS analysis is the inability to distinguish between various common metabolites with identical monoisotopic masses, such as several carbohydrates (Tables S1 and S2). This was partially addressed by analyzing analytical standards for selected carbohydrates applying GC/MS. Infected sprouts had decreased levels of D-fructose (−28.01%) and myo-inositol (−22.47%) whereas a general increase in the remaining carbohydrates was observed (Table S1).

### *Rhizoctonia solani* infection alters the content of sprouts in phenolics, cell wall-bound amides, and nucleosides

Sprout's response to pathogen invasion resulted in substantial alterations in their phenolics content (Fig. 3c and Table S1). Among the identified metabolites, the level of ferulic acid marked the highest increase (22.34%) whereas that of chlorogenic acid decreased after infection. In addition to the regulation of phenolics pathway, increased biosynthesis of *α*-tocotrienol (vitamin E fraction) was observed in infected sporuts. Other compounds implicated in plant-microbe interactions are the cell wall-bound tyramine amide *N*-feruloyltyramine and adenine which also increased considerably. On the other hand, compounds such the putrescine amide *N*-feruloylputrescine showed a substantial decrease after infection.

### Phenylacetic acid (PAA) is involved in pathogenesis of *Rhizoctonia solani* in potato sprouts

Using the in-house built target library for *R. solani*, phenylacetic acid (PAA) was tentatively identified in FT-ICR/MS (ESI^−^) spectra with relative peak intensity of 0.19% (data not shown). The presence of this phytotoxic metabolite in the infected sprouts only, which is among the most extensively studied phytotoxic metabolites of *R. solani* [31], [32] further supports its role in pathogenesis.

### *In vitro* bioactivity of biomarkers of potato sprout defense against *Rhizoctonia solani*

The exploitation of metabolomics for the discovery of plant-derived metabolites with potential as new sources of bioactivity has been recently introduced [13]. The antifungal activity of selected biomarkers of plant defense, including *α*-solanine, was assessed against *R. solani*. Based on the EC_50_ values, *α*-solanine and cinnamic acid were the most bioactive plant-derived metabolites with bioactivity significantly lower (*P*<0.05) than that of the reference fungicide azoxystrobin (Table S3).

### Metabolomics networking provides a global overview of the fluctuation of potato sprout's metabolome in response to *Rhizoctonia solani* infection

One of the major goals of the study is to introduce a robust pipeline for the integration of metabolomics data into the global metabolome of potato sprouts, providing a complete mapping of its perturbation in response to pathogen infection, and at the same time to highlight pathways and corresponding conceptual key enzymes and genes of plant's defence *in silico* (Table S4). Perturbations of sprout's metabolome and selected sub-networks in response to pathogen invasion were visualized using the software Cytoscape (Fig. 4). For the construction of the network, the PotatoCyc database was used, which had been previously reconstructed and curated based on results of our analyses, on-line databases, and information from the literature. Gaps of the extracted sub-networks were filled as previously suggested [33]. An exhaustive list of these enzymes and genes is presented in Table S4. Briefly, the up-regulated metabolites in the infected sprouts, belonging to 40 biosynthetic pathways and super-pathways, were conceptually linked to 107 enzymes and 222 corresponding encoding genes.

## Discussion

### Alterations in biosynthesis of alkaloids in *Rhizoctonia solani* infected sprouts

Injury of a plant cell and the presence of fungal-derived metabolites cause alterations in surrounding cells leading to systemic responses. Mevalonic acid and deoxy-xylulose pathways played a central role in sprout's defense by regulating the biosynthesis of glycoalkaloids and phytoalexins following *R. solani* invasion. Both pathways were activated resulting in increased concentrations of glycoalkaloids which are known to participate in the defense mechanism against fungal attacks [34], [35].

Based on established knowledge on the synergistic action between the *α*-chaconine and *α*-solanine [16], it is noteworthy mentioning the drop in the ratio of *α*-chaconine to *α*-solanine from 2.70% in uninfected to 2.35% in infected sprouts. These metabolites exert their toxicity by causing loss of membrane integrity due to the formation of complexes with membrane sterols. Bioactivity tests showed that *α*-solanine is toxic to *R. solani* with a half maximal effective concentration (EC_50_) of 0.19 M (Table S3). However, successful pathogens avoid such toxicity by removing sugar chains from the molecule of glycoalkaloids (i.e., hydrolysis) or by pH alteration [34]. Taken together, the hydrolytic degradation of *α*-solanine and *α*-chaconine to their catabolic and less toxic *β*- and *γ*- forms [36] during sprout colonization by *R. solani* (Figure S4) is a plausible scenario. It seems that this process in combination with the increased resistance of *R. solani* to sprout glycoalkaloids [37] are key elements contributing to the success of *R. solani* to overcome their toxicity. Although not yet assessed in *R. solani*, a rhamnosidase and *α*-chaconinase purified from potato fungal pathogens were able to convert *α*-chaconine to *β*-chaconine via a stepwise removal of a sugar unit from the trisaccharide chain, rendering the metabolite less bioactive [38], [39]. To what extent does *R. solani* metabolize/hydrolyze *in vivo* the two potato glycoalkaloids still awaits investigation. The progression of the disease, as it is expressed by the development of necrotic lesions, in the presence of increased levels of *β*- and *γ*- chaconine and solanine confirms that *R. solani* is tolerant to these glycoalkaloids, highly suggesting that resistance to glycoalkaloids is prerequisite for infection, which has been previously demonstrated in glycoalkaloid-containing plants attacked by pathogens [37]. Although our study proposes the hydrolytic degradation of *α*-chaconine and *α*-solanine, *in vivo* experiments are further required to confirm our observation. However, the hydrolytic degradation of potato glycoalkaloids could partially explain the fair correlation between their content and resistance against fungal pathogens [40], [41].

Similarly to solanidine-derived, solasodine-derived glycoalkaloids exhibit membrane-disrupting properties [42]. Their appearance and large increase in infected sprouts could be plausibly linked to solasodine degradation via a similar mechanism to that operating during *α*-solanine and *α*-chaconine degradation, or increased biosynthesis. *In vitro* studies have shown that solasonine is ineffective against *R. solani* hyphae [43]. This branch of potato steroidal alkaloids biosynthesis is largely unknown, and our data suggest its involvement in potato-pathogen interaction.

The production of phytoalexins in response to attack of plant tissues by a variety organisms or exposure to elicitors is well documented [44], [45], [46]. Since they are known to be bioactive against various potato pathogens [44], [45], [47], it is highly probable that their accumulation at the site of sprout's infection effectively limits or delays the spread of *R. solani*. Our study represents the first report on the biosynthesis of potato sprout phytoalexins in response to *R. solani* infection.

The role of the glycosidase inhibitors calystegines in plant physiology has not been fully elucidated, and their involvement in plant defense remains elusive. Intake of calystegines by insects designates them as defense substances allowing only adapted insects to feed on plants rich in calystegines [48]. Our study showed that sprout's calystegines do not play a role in their defense against *R. solani* and thus, it seems that they represent a stimulus-specific plant defense response, an observation that merits further investigation.

### *Rhizoctonia solani* affects fatty acid and fatty acid hydroperoxides metabolism

Results indicate involvement of FAs, mainly unsaturated, and oxylipins in potato sprout-*R. solani* interactions. Reduced content of oleic acid (C18:1) has been associated with induction of plant resistance against pathogens via the stimulation of multiple *R* genes transcription [49]. On the other hand, divinyl ether FAs are synthesized via the lipoxygenase-mediated biosynthetic pathway from linoleic and linolenic acids, and their role as phytoalexins and participation in plant defense has been illustrated in solanaceous crops upon infection with microbial pathogens, notably oomycetes [50], [51], observations which are in agreement to our findings.

Oxidized FAs are generated enzymatically or non-enzymatically in stressed plants [52], are fungitoxic [53], [54] and act as precursors of wound-inducible signals [55]. Involvement of oxidative reactions in potato leaves [56] after infection by *Phytophthora infestans* or treatment of potato tuber disks with *P. infestans* elicitors [57] through activation of lipoxygenases (LOXs) has been reported. The role of linoleic and linolenic acids hydroxides as endogenous elicitors of phytoalexin production is well documented in rice (*Oryza sativa*)-*Pyricularia oryzae* pathosystem [58]. Additionally, *ω*-hydroxyacids, mainly hydroxyoleic acid, are the major monomers composing the polyaliphatic domain of the biopolymer suberin which is the major component of the epidermal tissues of sprouts. Thus, the increased content of hydroxyoleic acid in infected sprouts is likely the result of suberin hydrolysis by *R. solani* enzymes or increased biosynthesis by the sprouts to repair ruptures of their suberin layer. Finally, although hydroperoxylinoleic acid is fungitoxic and has been reported to increase in plants in response to fungal infection [54], its decrease in infected sprouts could be partially attributed to the increased biosynthesis of colneleic acid via the activation of the enzyme 9-divinyl ether synthase (StDES) (Fig. 5). This notion is supported by the observations of elicitor-treated potato cells infected with *Phytopthora infestans* or infiltrated with *Pseudomonas syringae* [59].

### Changes in carboxylic acid content of infected sprouts

Carboxylic acids have multiple biological functions in plants, with recent reports to implicate some of them in pathogenesis. The increase in the content of infected sprouts in azelaic acid is indicative of its implication in sprout-*R. solani* interactions. This observation is in agreement with results of a recent study implicating azelate to systemic acquired resistance (SAR) in *Arabidopsis* to *Pseudomonas syringae* [60]. Similarly, oxalic acid triggers hypersensitive response (HR) in sunflower, in a reaction catalysed by oxalate oxidase (OXO) resulting in the production of H_2_O_2_, which in high concentrations is fungitoxic and strengthens plant defense [61]. Such fungitoxicity was recorded in our *in vitro* experiments (Table S3). The increase in the levels of gluconic and *α*-keto-*d*-gluconic acids of infected sprouts is indicative of the activation of pentose phosphate pathway which is a process of glucose turnover leading to the production of fundamental constituents of nucleotides such as NADPH and pentoses. Additionally, *α*-keto-*d*-gluconic acid is bioactive [62] indicating its possible role in potato sprout defense mechanism. Although galacturonic acid has been reported as an endogenous suppressor of disease resistance reaction of wheat against *Puccinia graminis* [63], it seems that these metabolites do not play such a physiological role during potato-*R. solani* interaction. Furthermore, although increased plant hormone levels trigger the expression of PR genes and activate plant defense responses in plant-pathogen pathosystems [64], the observed decrease in indolebutyric acid indicates that this hormone does not play a role in the defense mechanism of potato sprouts against *R. solani*.

### Fluctuations in the amino acid pool of infected sprouts

The decrease in the protein amino acid pool following infection indicates their utilization via the stimulation of pathogen-related (PR) protein and metabolite biosynthesis of sprouts in response to the invading pathogen. The observed increase of pyroglutamic acid is plausible to occur in order for sprouts to support the biosynthesis of PR proteins containing pyroglutamic acid at their N-terminus, since the metabolite acid plays a key role in stabilizing or mediating PR protein and peptide structures in plant-pathogen pathosystems [65].

On the other hand, non-protein amino acids are homologs of protein amino acids, however they do not incorporate into proteins and their physiological role is still largely unknown [66]. Although the origin of *β*-alanine has not been elucidated yet, its accumulation likely represents a response of infected sprouts in order to sustain increased biosynthesis of the coenzyme A (CoA) which plays a central role in various metabolite biosyntheses [67]. GABA is found in animals and plants and it is synthesized through *α*-decarboxylation of L-glutamic acid in a reaction catalyzed by L-glutamic acid decarboxylase (GAD, E.C. 4.1.1.15). GAD activity is triggered by increased H^+^ and Ca^+^ levels in the cytoplasm leading to GABA biosynthesis which is important for pH regulation and plants' physiology [68]. Recent evidence established that GABA accumulates in plants in response to various abiotic and biotic stresses [12], [68], [69] and suggests that GABA accumulation is a rapidly induced, local resistance mechanism and may participate in protection against reactive oxygen species. These findings support the implication of GABA in potato defense mechanism against *R. solani*. Modifications in regulatory properties and fluxes in metabolic pathways are observed in osmotically stressed plants. Osmotically-stressed *Brassica napus* accumulate pipecolic acid via lysine catabolism catalyzed by lysine-ketoglutarate reductase (LKR, E.C. 1.5.1.8) and saccharopine dehydrogenase (SDH, E.C. 1.5.1.9) [70]. Taken together, it is highly likely that increased biosynthesis of the osmo-protectant pipecolic acid in infected tissues is the sprout's response to the osmotic stress caused by the ruptures of cell membranes.

### Alterations of the content of infected spouts in carbohydrates

Fluctuation in the levels of carbohydrates of infected sprouts is indicative of a general remobilization of sugars in response to pathogen invasion. Decreased levels of carbohydrates have been reported as a plant response to fungal invasion [71]. The decreased levels of D-fructose in infected sprouts could be attributed to its catabolism for energy generation and biosyntheses of secondary metabolites. It is noteworthy to mention that clear discrimination between fungal-derived and host-derived carbohydrates is not apparent as many of these carbohydrates such as *α*,*α*-trehalose, detected in increased levels are also fungal components (Table S2 and [31], [72]). Nonetheless, increased levels of trehalose have been also reported in *Arabidopsis thaliana* leaves infected with *Pseudomonas syringae* [12].

### *Rhizoctonia solani* affects the phenolic and cell wall-bound amide contents of sprouts

Results revealed the implication of several phenolics and cell wall-bound amides as responses of sprouts to fungal invasion. Ferulic acid is known to exhibit antifungal and antioxidant properties [73] and is a key component of suberization [22]. Therefore its increase is likely to represent a response of the biosynthetic mechanism of sprouts to the invading pathogen aiming to limit its progress into plant tissues. In contrast, the observed decrease in chlorogenic acid is in agreement with previous study on the response of potato cells to elicitors [74], indicating limited involvement in pathogenesis. On the other hand, the antioxidant mechanism of infected plant tissues was strengthened by the increased biosynthesis of *α*-tocotrienol (vitamin E fraction), which is an effective inhibitor of lipid oxidation [75].

Our study also highlighted the involvement of cell wall-bound amides in pathogenesis. High levels of *N*-feruloyltyramine have been also reported in potato leaves and cell cultures in response to *P. infestans* infection and treatments with elicitors, respectively [74], [76]. Collectively, the metabolite plays a role in sprout defense through its incorporation into plant cell walls, producing a suberin-like polymer that increases its resistance against pathogen-excreted enzymes [77], [78]. Additionally, adenine, is a nucleoside implicated in multiple cell processes such as protein synthesis through participation in DNA and RNA molecules, and the biosynthesis of adenylates (i.e. ATP, ADP, and AMP) through the regulation of adenylate pools [79]. The observed decrease in the levels of *N*-feruloylputrescine following pathogen attack is in agreement with the recent finding on elicitor-treated potato tubers [74].

### Metabolomics networking provides a global overview of the fluctuation of potato sprout metabolome in response to *Rhizoctonia solani* infection

To date, the majority of metabolomics studies have been limited to a listing of biomarkers without their further incorporation into the global metabolic network of the biological system being studied. Therefore, in an effort to place metabolomics within the context of systems biology, a high-throughput metabolomics protocol was developed. Such data-driven computational approach enabled tracing of individual metabolic processes and the mapping of signature metabolites within the potato sprout metabolome. Furthermore, reactions of the potato's metabolic network were conceptually connected via enzymes catalyzing the biosynthesis of detected signature metabolites and encoding genes *in silico*.

### Conclusion

It is now well recognized that a combination of analyzers is required to improve coverage of the analysed metabolomes. This study has used GC/MS and FT-ICR/MS to monitor fluctuations in metabolite composition in potato sprouts under the influence of a fungal pathogen. The applied bioanalytical and bioinformatics protocols enabled the mapping of a significant portion of potato sprout metabolome and provided complementary data for its deconvolution, which are prerequisites for standardized high-throughput metabolomics. Additionally, the robust visualization and mining of global metabolite networks using bioinformatics software proved to be a powerful approach for their classification and the detection of biomarkers that substantiate our hypothesis that the metabolomes of healthy and infected potato sprouts are substantially different. *R. solani* early invasion of potato sprout tissues triggers a general disturbance of their metabolism leading to complex responses. Among the induced metabolites, glycoalkaloids and phenolics are well-known pathogen-induced metabolites of Solanaceae [16]. Results also revealed a general disturbance of anabolic and/or catabolic plant processes causing substantial fluctuations in the content of a large number of metabolites belonging to amino, carboxylic, and fatty acids, some of which are key components of HR, SAR, and other defense-related responses, such as the activation of LOXs. Furthermore, analyses highlighted potential targets at the genome and proteome level for further research and identified several antifungal plant-derived metabolites which are amendable as biomarkers in biomarker-assisted crop breeding or could be used *per se* or as lead structures for the development of new crop protection agents.

## Materials and Methods

### Chemicals and Reagents

All chemicals and reagents were of the highest available purity. Pyridine, methoxylamine hydrochloride, *N*-methyl-*N*-(trimethyl-silyl)trifluoroacetamide (MSTFA) for GC/MS analyses, formic, cinnamic, fumaric, succinic, and oxalic acids, and all analytical standards were purchased from Sigma-Aldrich Canada Ltd. (Oakville, ON). Ethanol, ethyl acetate, methanol, ammonium hydroxide and water (HPLC grade) were purchased from Fisher Scientific Company (Ottawa, ON). The fungicide azoxystrobin was a courtesy of Syngenta Crop Protection Canada, Inc (Guelph, ON).

### Biological material

Certified potato tubers (*S. tuberosum* var. Kennebek) were supplied by Bon Accord Elite Seed Potato Center (NB, Canada). Starter cultures of a highly pathogenic *R. solani* AG3 isolate 114 (M. Cubeta, North Carolina University, USA) were maintained on oat kernels at 4°C. Agar plugs from starter cultures were placed on potato dextrose agar (PDA; Difco Laboratories, MI, USA) in Petri plates (9 cm in diameter) and grown at 24°C in the dark.

### Sprout inoculation

Pre-sprouting of potato tubers occurred in growth chambers in the dark at 0°C with 90% relative humidity. In total, eight potato tubers that are healthy and uniform in size and overall appearance were selected and treatments were performed when sprout's length was 8.0 cm. The basal portion of sprouts (one sprout per tuber) was sandwiched between two PDA strips (2 cm×8 cm) of a five-day-old *R. solani* culture. Treatments with PDA strips alone served as controls. All treatments were performed under photosynthetically inactive black light (365 nm). Development of infection cushions and necrotic lesions on inoculated sprouts (Fig. 1) was monitored stereoscopically every 24 h. After 72 h, the PDA strips of all treatments were discarded and sprouts were harvested and prepared for metabolite extraction. This time point was specifically chosen in order to capture the onset of infection structures and development of necrotic lesions. Eight replications were performed per treatment.

### Sampling and extraction

The accuracy of sampling of plant tissues is critical for the study of plant-pathogen interactions applying metabolomics. To minimize errors and variation, uniform size portions (40×30 mm; 40 mg of fresh weight-f.w.) were taken from the edges of the developed necrotic lesions 72 h post-inoculation under a stereoscope (Fig. 1). Similar sprouts' portions were taken from the controls (Fig. 1b). Additionally, mycelia (20 mg f.w.) from 3-day-old *R. solani* cultures (Fig. 1a) were analyzed in order to identify fungal-derived metabolites that could possibly have leverage in the analyses. Increases in metabolites of infected plant tissues that are also highly abundant in *R. solani* endo-metabolome were not biologically interpreted (Table S1). Quenching of metabolism was performed by adding liquid N_2_, and samples were kept at −80°C until further use. Prior to extraction, samples were lyophilized for 24 h. Extraction was performed in Eppendorf tubes (2 ml) containing 200 mg of glass beads (5 mm in diameter, Biospec Products Inc.) and 1 ml of methanol∶ethyl acetate solution (50∶50, v/v) using a FastPrep (FP 120, Savant Instruments Inc, NY, USA) for 45 s at maximum speed (6.5 m sec^−1^). This was repeated three times. This solvent mixture has proved to be efficient for the extraction and profiling of primary metabolites such as amino acids, carbohydrates, carboxylic and fatty acids [31], [72]. Extracts were then filtered through 0.2 µm filters (Millex-FG, Millipore, MA, USA) and divided into two equal portions (0.5 ml) and placed into autosampler vials (2 ml) for GC/MS and FT-ICR/MS analyses. Extracts for FT-ICR/MS analyses were further divided into equal portions (0.25 ml) for analyses in ESI^+^ and ESI^−^ modes. All extracts were dried using a Speedvac® AES1010 (Savant Instruments Inc).

### Mass spectrometry analyses

Two different MS analyzers were used in this study; the GC/MS which covers mainly the primary metabolism and low molecular weight metabolites, and FT-ICR/MS which provides high accuracy (<1 ppm) and resolving power, thus enabling the accurate detection of primary as well as secondary metabolites. Because of their capabilities, the two platforms can complement each other, providing additional information which helps in the achievement of a broad coverage of plant metabolome and strengthen metabolite identification confidence.

### Gas chromatography/mass spectrometry analysis

Sample derivatization was performed as previously described [31]. Briefly, 80 µL of a methoxylamine hydrochloride solution (20 mg ml^−1^ in pyridine) was added in the dry extracts at 30°C for 120 min, followed by the addition of MSTFA (80 µL) at 37°C for 90 min. The derivatized samples were added in microinserters (150 µL, Fisher Scientific Company) into autosampler vials (2 ml) and analyzed with an Agilent 7890A GC platform (Agilent Technologies Inc. Santa Clara, CA, USA) coupled with a 5975C series mass selective detector (MSD) and a 7693A series autosampler. Electron ionization of 70 eV was used and the value of the electron multiplier was 1480 V throughout analyses. Full scan mass spectra were acquired at the mass range of 50 to 800 Da at 1 scan s^−1^ rate with a 10.0-min solvent delay. The temperature for the ion source was set to 150°C, for the transfer line to 230°C, and for the injector to 230°C. Aliquots of 1 µL were injected into a HP-5MS ultra inert (UI) capillary column (30 m×250 µm I.D., 0.25 µm film thickness; Agilent Technologies Inc.) and the injector split ratio was set to 10∶1. The initial temperature of the oven was 70°C stable for 5 min, followed by a 5°C min^−1^ increase to 310°C and finally stable for 1 min. Helium was used as the carrier gas at a constant flow rate of 1 ml min^−1^. Calibration of the instrument was performed daily throughout the course of the analyses using the default automatic calibration mode as recommended by the manufacturer.

Chromatogram acquisition, peak deconvolution, and MS library searches were performed using the Agilent MSD Chemstation version E.02.00.493. Peaks corresponding to column bleeding and reagent peaks were excluded from further analyses. Putative identification of metabolites was performed by matching their mass spectra to spectra in NIST 08 library (National Institute of Standards and Technology, Gaithersburg, MD, USA). The definitive identification of metabolites was based on matching their mass spectra and retention times (RT) to those of the authentic chemical standards analyzed on the same platform with the same analytical method [7].

Pre-processing of total ion chromatograms (TIC) such as baseline correction, alignment, peak picking, and integration were performed using the ACD/Spec Manager v.12.00 (Advanced Chemistry Development, Inc., ACD/Labs, Toronto, Canada). Data were exported as “.txt” files to MS Excel® for the creation of data matrices.

### Fourier transform ion cyclotron resonance/mass spectrometry analysis

Analyses were performed using an IonSpec Explorer FT-ICR/MS (IonSpec Inc., Lake Forest, CA, USA) and all experimental events were controlled using the Omega8 software (IonSpec Inc.). The analyzer was equipped with a Z-spray source (Waters Corporation) a quadrupole ion guide, a standard cylindrical ion cyclotron resonance (ICR) cell, and an actively shielded superconducting magnet of 7-Tesla. For ESI^+^ the potential on the electrospray emitters was set to 3.0 kV whereas for ESI^−^ to −3.0 kV. For analyses in ESI^+^ and ESI^−^ modes, 0.15 ml of a mixture of methanol∶formic acid (0.1% v/v) (50-50, v/v) or methanol∶ammonium hydroxide (0.1%, v/v) (50-50, v/v) were added to the dried samples, respectively. Extracts were then transferred in microinserters (150 µL) into autosampler vials (2 ml). Samples were directly infused at a flow rate of 0.5 to 1.0 µl min^−1^ through a 100 µl syringe (Hamilton, Reno, NV, USA) and analyses were performed at a resolution of 100,000 (full width at half maximum, FWHM). Spectra were acquired over the range of 100–1,000 Da using the SIM-stitching method which has been shown to increase the dynamic range without compromising mass accuracy [80]. In order to optimize the performance of the instrument, calibration was performed daily using solutions for ESI^+^ and ESI^−^ recommended by the manufacturer. In total, eight biological replicates were performed per treatment with two additional technical replicates for monitoring instrument performance.

For the deconvolution of spectra and the putative identification of metabolites, searches were initially performed using the metabolite-species database KNApSAcK (http://kanaya.aist-nara.ac.jp/KNApSAcK/) for the most common ionization forms in ESI^+^ and ESI^−^ within a mass error of less than 1.5 ppm (*Δppm*<1.5). Possible molecular forms for candidate neutral forms were calculated using an elemental composition calculator (www.wsearch.com.au) according to proposed guidelines [81]. In a second step, the identity of the tentatively identified metabolites was cross-validated using data from GC/MS analyses. In addition to KNApSAcK, searches were performed against an in-house built library of potato metabolites, composed of accurate masses of metabolites reported to be present in potato, potato sprouts, *R. solani* endo- and exo-metabolome, or produced during potato interactions with pathogens. Data for the construction of the library were acquired from public available databases PotatoCyc (http://pathway.gramene.org/gramene/potatocyc.shtml), PlantCyc database (http://www.plantcyc.org/), Chemspider (http://www.chemspider.com/PropertiesSearch.aspx), Kyoto Encyclopedia of Genes and Genomes LIGAND (http://www.genome.jp/kegg/ligand.html), and Metlin (http://metlin.scripps.edu/).

Raw FT-ICR/MS data were exported as text files (*.txt) and imported into MS Excel® for the construction of data matrices. Ions not related to the biological material were excluded from further analyses. Ions corresponding to isotopes (M+1, M+2, M+3) and/or ions corresponding to several ionization forms of the same metabolite were summed. Additionally, based on mass detection errors, spectra were aligned. As a rule, ions were considered to represent the same metabolite if they occurred within a 0.5 ppm spread along the *m/z* axis for the different spectra. Also, ions with less than 50% abundance within replications of the same treatment were removed in order to strengthen the uniformity of data.

### Statistical analyses

Data matrices were subjected to multivariate analyses using the SIMCA-P+ v.12.0 software (Umetrics, MKS Instruments Inc., Andover, MA, USA). Initially, PCA was performed for the evaluation of data and detection of outliers. The discovery of biomarkers was based on PLS-DA regression coefficients (*P*<0.05) since by applying PCA, it is not certain that the computed principal components (PCs) represent the largest sources of variation [82]. Standard errors were calculated using Jack-knifing which is based on the variability in the model parameters encountered in the different cross-validation cycles with 95% confidence interval [83]. The performance of the models was assessed by the cumulative fraction of the total variation of the *X*'s that could be predicted by the extracted components [*Q^2^_(cum)_*] and the fraction of the sum of squares of all *X*'s (*R^2^X*) and *Y*'s (*R^2^Y*) explained by the current component.

Two-dimensional hierarchical cluster analysis (2D-HCA) and heatmaps were performed using the software MATLAB (v.R2011b, The MathWorks Inc., Natick, MA, USA). Heatmaps illustrate alterations (fold change) in the concentration of metabolites between treatments encoded in a colour-code. Cluster distances were calculated using the Ward's linkage method.

### Metabolomics network analysis and visualization and linking between metabolome, proteome and genome

Potato metabolome was visualized using the bioinformatics software Cytoscape (v.2.8.2., http://www.cytoscape.org/) [84] and the reconstructed and curated based on our analyses PotatoCyc database (http://pathway.gramene.org/gramene/potatocyc.shtml). Data were also acquired from PlantCyc (http://www.plantcyc.org/), KEGG (http://www.genome.jp/kegg/) databases, and the literature. Based on results of metabolomics analyses and the abovementioned sources, enzymes catalyzing the biosynthesis of biomarkers of plant response to the pathogen attack and corresponding encoding genes were inferred and highlighted.

### Assessment of fungitoxicity of selected biomarkers of plant response to the pathogen

For the assessment of the fungitoxicity of selected biomarkers of potato defence against *Rhizoctonia solani*, stock solutions in ethanol were used. The inhibition of radial fungal growth on PDA was used to assess the bioactivity of the compounds. Radial growth in two different directions was measured. Amounts of stock solutions were added to 100 ml of PDA in 250 ml screw-cap bottles to obtain final concentrations of 400, 200, 100, 50, 25 and 12.5 ppm. PDA with appropriate concentrations in ethanol was used as control. Plates were inoculated with a 5 mm in diameter plug of one-week old *R. solani* culture. Assessment of half maximal effective concentration (EC_50_) was recorded 96 h after inoculations of the plates. EC_50_ was designated as the concentration (molarity, M) of the applied compound that caused 50% inhibition in the mycelial radial growth of the fungus. The experiment was repeated three times with three replications per treatment.

### Quality control of metabolomics analyses

All experimental steps were performed following standard operating procedures (SOP) and quality control (QC) measures. In order to evaluate the presence of compounds not related to the analyzed biological material (i.e., contamination during sample preparation, column bleeding, solvent impurities, or instrument contamination), blank samples prepared following identical protocols to those applied for biological samples were run alongside the experimental samples. Furthermore, two technical replications were performed for randomly selected samples per treatment and solutions of analytical standards were analyzed for the assessment of instrument's performance. Internal calibration of data for the estimation of mass errors was performed using accurate masses of identified common metabolites such as amino acids (ESI^+^) and lipid acids (ESI^−^).

## Supporting Information

Figure S1 **Representative FT-ICR/MS and GC/MS spectra of control and infected potato sprouts by** ***Rhizoctonia solani*** **.**
(TIFF)

Figure S2 **Principal component analysis PC1/PC2 score plots of FT-ICR/MS metabolic profiles recorded in positive (a) and negative (b) modes, GC/MS metabolic profiles (c), and combined FT-ICR/MS and GC/MS metabolic profiles (d) of healthy (▴) and** ***Rhizoctonia solani*** **infected (▪) potato sprouts.**
(TIFF)

Figure S3 **Two-dimensional hierarchical cluster analyses and heatmaps of FT-ICR/MS metabolic profiles recorded in positive (a) and negative (b) modes, GC/MS metabolic profiles (c), and combined FT-ICR/MS and GC/MS metabolic profiles (d) of healthy (▴) and** ***Rhizoctonia solani*** **infected (▪) potato sprouts.**
(TIFF)

Figure S4 **Hydrolytic degradation of** ***α*** **-chaconine and** ***α*** **-solanine to solanidine.**
(TIFF)

Table S1 **Changes in potato sprouts' metabolome in response to** ***Rhizoctonia solani*** **72 h post infection.**
(PDF)

Table S2 **Identified metabolites of eight-day-old mycelia of** ***Rhizoctonia solani*** **AG3 grown on PDA that could possibly have a high leverage during the biological interpretation of metabolomics data.**
(PDF)

Table S3 **Half maximal effective concentration (EC_50_) of selected antifungal potato biomarkers against** ***Rhizoctonia solani*** **.**
(PDF)

Table S4 **Potato sprouts' metabolites with the highest fluctuation 72 h after infection by** ***Rhizoctonia solani*** **with enzymes catalyzing their biosynthesis and corresponding encoding genes.**
(PDF)
