# Supplementary figures and images for: FT-ICR/MS and GC-EI/MS Metabolomics Networking Unravels Global Potato Sprout's Responses to Rhizoctonia solani Infection
Source: PLoS One. 2012 Aug 3;7(8):e42576. doi: 10.1371/journal.pone.0042576 (PMC3411821; doi:10.1371/journal.pone.0042576)

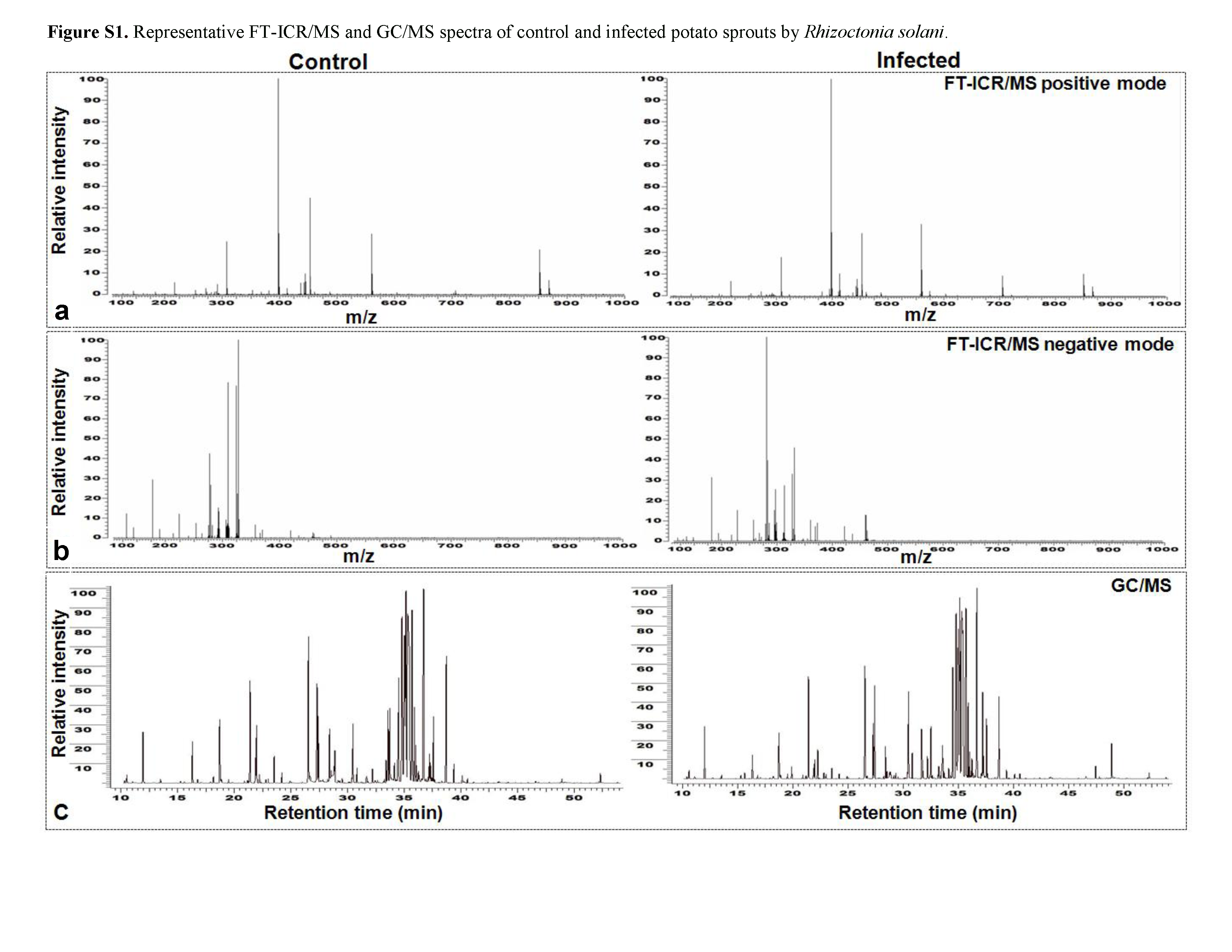

Supplement: Figure S1 — Representative FT-ICR/MS and GC/MS spectra of control and infected potato sprouts by Rhizoctonia solani . (TIFF) [file pone.0042576.s001.tiff]

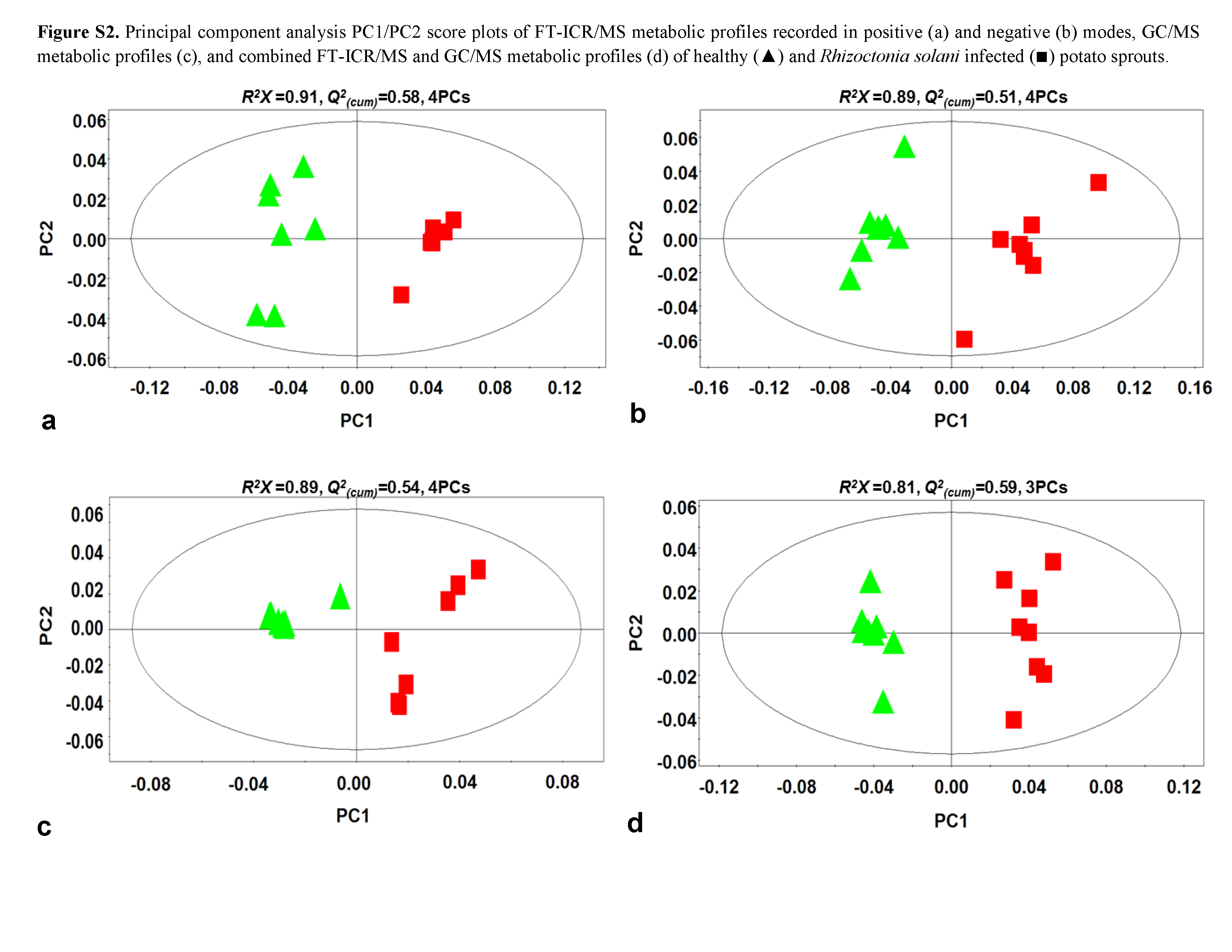

Supplement: Figure S2 — Principal component analysis PC1/PC2 score plots of FT-ICR/MS metabolic profiles recorded in positive (a) and negative (b) modes, GC/MS metabolic profiles (c), and combined FT-ICR/MS and GC/MS metabolic profiles (d) of healthy (▴) and Rhizoctonia solani infected (▪) potato sprouts. (TIFF) [file pone.0042576.s002.tiff]

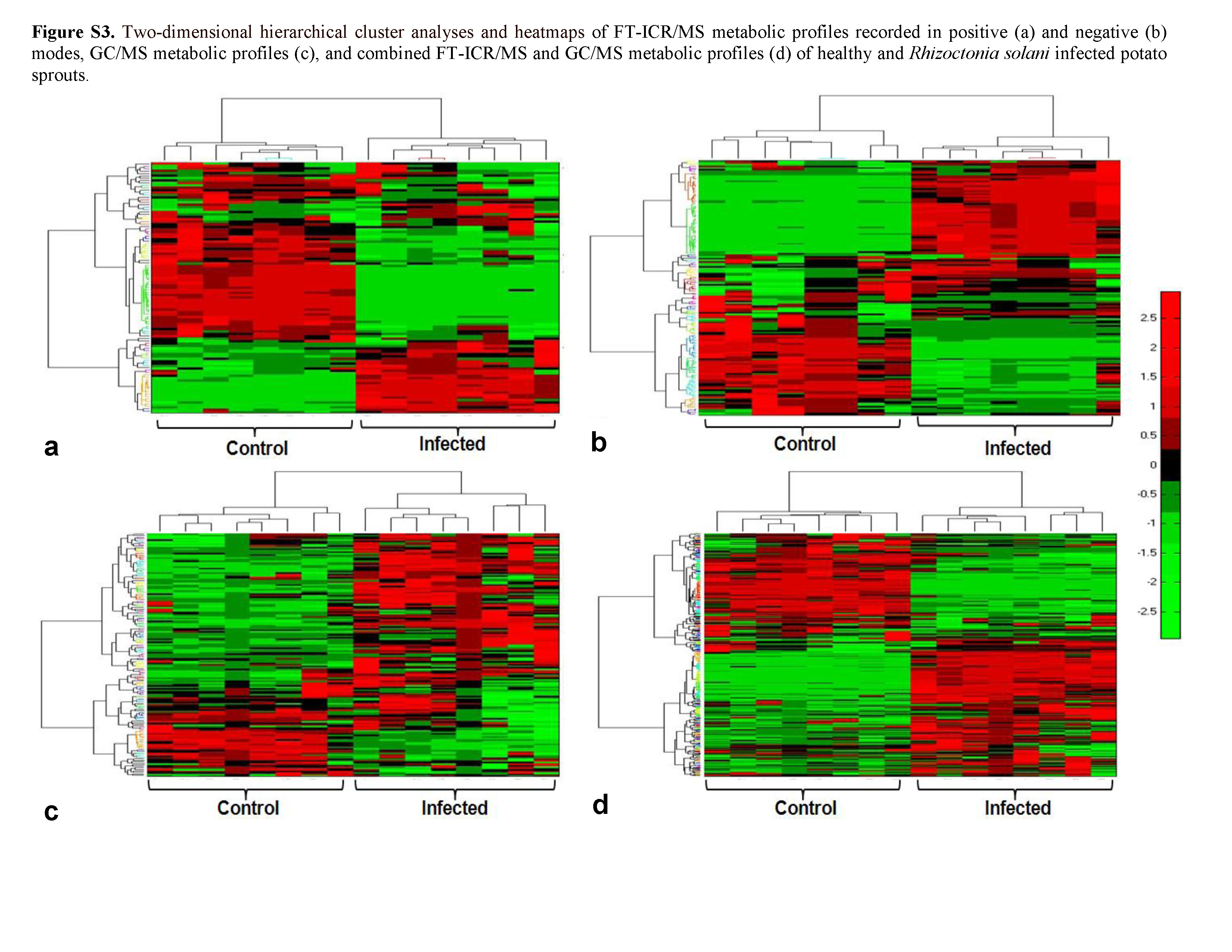

Supplement: Figure S3 — Two-dimensional hierarchical cluster analyses and heatmaps of FT-ICR/MS metabolic profiles recorded in positive (a) and negative (b) modes, GC/MS metabolic profiles (c), and combined FT-ICR/MS and GC/MS metabolic profiles (d) of healthy (▴) and Rhizoctonia solani infected (▪) potato sprouts. (TIFF) [file pone.0042576.s003.tiff]

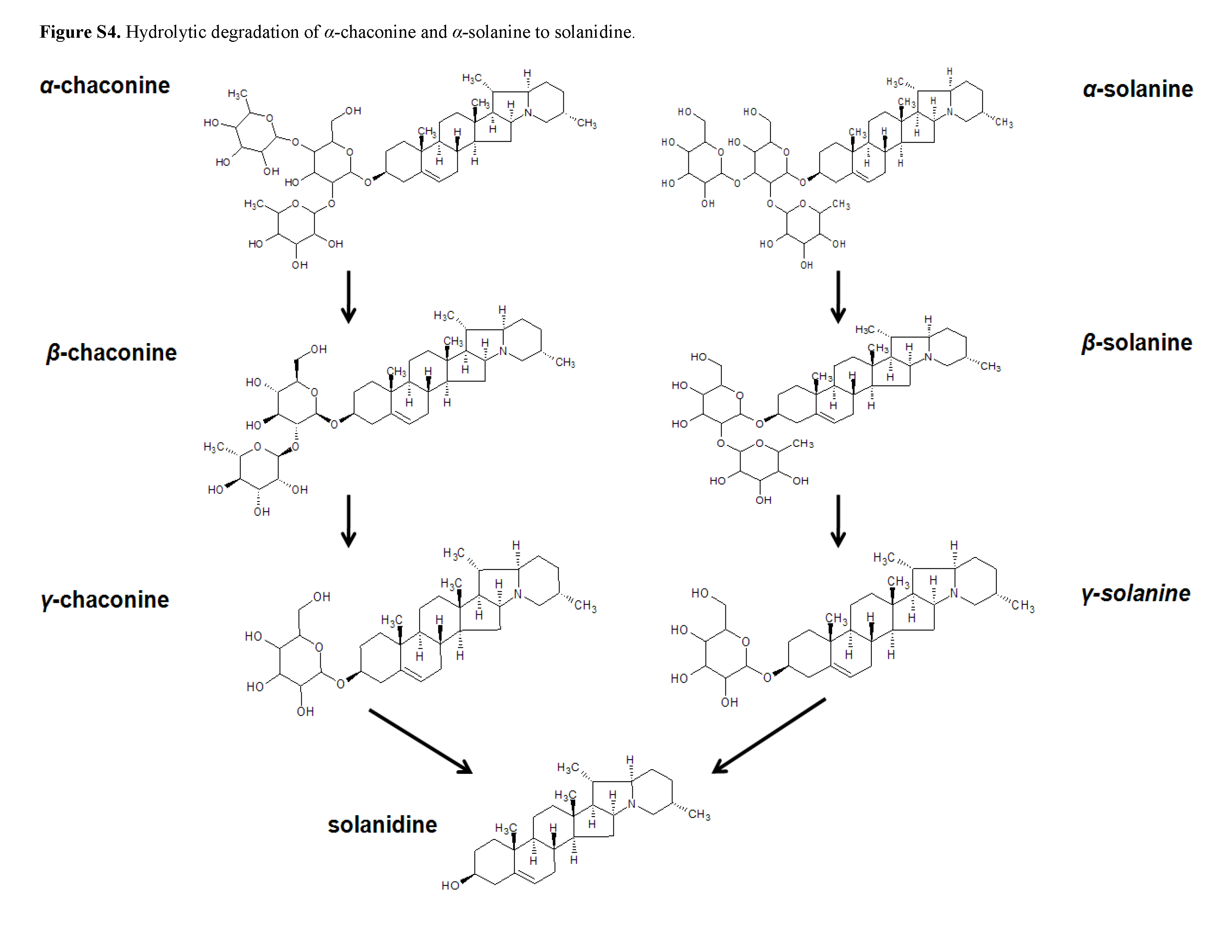

Supplement: Figure S4 — Hydrolytic degradation of α -chaconine and α -solanine to solanidine. (TIFF) [file pone.0042576.s004.tiff]
