# Supplementary material for: FT-ICR/MS and GC-EI/MS Metabolomics Networking Unravels Global Potato Sprout's Responses to Rhizoctonia solani Infection
Source: PLoS One. 2012 Aug 3;7(8):e42576. doi: 10.1371/journal.pone.0042576 (PMC3411821; doi:10.1371/journal.pone.0042576)
Supplement: Table S1 — Changes in potato sprouts' metabolome in response to Rhizoctonia solani 72 h post infection. (PDF) [file pone.0042576.s005.pdf]

Table S1. Changes in potato sprouts' metabolome in response to *Rhizoctonia solani* 72 h post infection. Metabolites with the largest variation in their relative content between control and infected sprouts and essential components of plant defence are displayed. Compound identification (ID) is provided according to the PubChem database (http://pubchem.ncbi.nlm.nih.gov/).

Metabolites	Relative content change in infected sprouts (%)[a]	Relative content of infected sprouts (%)[a]	Analytical platform[b]	Molecular form	Monoisotopic mass (Da)	*P*-values	ID
Alkaloids							
Sesquiterpene alkaloids							
Phytuberin	*n/a*[c]	0.20	FT+	$C_{17}H_{26}O_4$	294.1831	*n/a*	10379702, 315114
Rishitin	*n/a*[c]	0.16	FT+	$C_{14}H_{22}O_2$	222.1619	*n/a*	108064, 534260
Solavetivone	*n/a*[c]	1.52	FT-	$C_{15}H_{22}O$	218.1671	*n/a*	10104810, 442399, 521550, 185777, 11085279
Steroidal alkaloids							
α-Chaconine	-36.82	4.78	FT+	$C_{45}H_{73}NO_{14}$	851.5031	<0.0001	104750, 442971
α-Solanine	-27.81	2.02	FT+	$C_{45}H_{73}NO_{15}$	867.4980	0.0022	30185, 51020424, 9549171, 262500, 6537493
β-Chaconine	88.91	0.95	FT+	$C_{39}H_{63}NO_{10}$	705.4452	<0.0001	119393
β-Solanine	*n/a*[c]	0.33	FT+	$C_{39}H_{63}NO_{11}$	721.4401	*n/a*	45479590

γ-Chaconine/*γ*-Solanine	57.07	7.64	FT+	$C_{33}H_{53}NO_6$	559.3873	<0.0001	160496/25245074
Solanaviol	*n/a*[c]	0.30	FT+	$C_{27}H_{43}NO_3$	429.3243	*n/a*	-
Solanidine	53.86	30.59	FT+	$C_{27}H_{43}NO$	397.3345		65727
Solasodenone	*n/a*[c]	0.65	FT+	$C_{27}H_{41}NO_2$	411.3137	*n/a*	331777
Solasodiene	*n/a*[c]	1.17	FT+	$C_{27}H_{41}NO$	395.3188	*n/a*	495009
Solasodine	88.81	3.89	FT+	$C_{27}H_{43}NO_2$	413.3294	<0.0001	5250, 31342, 442985, 5200420, 44584207, 6432036, 5701987
Solasonine	*n/a*[c]	0.06	FT+	$C_{45}H_{73}NO_{16}$	883.4929	*n/a*	73410, 119247, 255983, 537159, 9876101, 9942086, 6326050
Solaspiralidine	*n/a*[c]	0.11	FT+	$C_{27}H_{41}NO_3$	427.3086	*n/a*	-
Nortropane alkaloids							
Calystegine A3	-49.34	0.05	FT+	$C_7H_{13}NO_3$	159.0895	<0.0001	442999, 183073, 45109777
Calystegine B2	-23.45	0.05	FT+	$C_7H_{13}NO_4$	175.0845	0.0009	124434, 443000
Amino acids							
Protein amino acids							
5-Oxo-L-proline (Pyroglutamic acid)	40.18	1.07	GC	$C_5H_7NO_3$	129.0426	0.043	7405
L-asparagine [d]	-32.32	3.21	GC	$C_4H_8N_2O_3$	132.0535	<0.0001	6267
L-glutamine [d]	-57.23	0.62	GC	$C_5H_{10}N_2O_3$	146.0691	0.0005	5961
L-histidine	-89.74	0.09	FT-	$C_6H_9N_3O_2$	155.0695	<0.0001	6274

L-isoleucine [d]	-43.52	0.46	GC	$C_6H_{13}NO_2$	131.0946	<0.0001	6306
L-leucine [d]	-59.25	0.15	GC	$C_6H_{13}NO_2$	131.0946	<0.0001	6106
L-proline [d]	-42.87	6.53	GC	$C_5H_9NO_2$	115.0633	<0.0001	145742
	-79.17	0.18	FT+			0.0118	
L-serine[d]	-34.43	0.68	GC	$C_3H_7NO_3$	105.0426	<0.0001	5951
L-threonine [d]	-48.43	0.58	GC	$C_4H_9NO_3$	119.0582	<0.0001	6288
L-valine	-68.56	0.07	FT+	$C_5H_{11}NO_2$	117.0790	<0.0001	6287
	-15.80	1.92	GC			0.0154	
Nonprotein amino acids							
β-Alanine [d]	20.05	0.05	GC	$C_3H_7NO_2$	89.0477	0.0246	239
4-aminobutanoic acid (GABA)[d]	20.87	3.67	GC	$C_4H_9NO_2$	103.0633	0.0205	119
Piperidine-*2*-carboxylic acid (Pipecolic acid)	33.18	0.02	GC	$C_6H_{11}NO_2$	129.0789	0.0058	849, 439227
CARBOHYDRATES							
D-Fructose [d]	-28.01	6.41	GC	$C_6H_{12}O_6$	180.0634	0.0216	5984
Myo-inositol [d]	-22.47	4.40	GC	$C_6H_{12}O_6$	180.0634	0.0050	892
Carboxylic acids and phenolics							
2-Hydroxybutanedioic acid (Malic acid)	-71.13	0.10	FT-	$C_4H_6O_5$	134.0215	0.0039	222656
	-21.52	6.64	GC			<0.0001	
2-Hydroxyundecanoic acid	-62.67	0.36	GC	$C_{11}H_{22}O_3$	202.1569		5282899
2-Thiobarbituric acid	20.9	5.96	GC	$C_4H_4N_2O_2S$	143.9993	0.0490	2723628
Citramalic acid	92.75	0.25	FT-	$C_5H_8O_5$	148.0372	<0.0001	1081
Chlorogenic acid	-95.03	0.01	FT-	$C_{16}H_{18}O_9$	354.0950	<0.0001	1794427
D-galacturonic acid	-21.58	0.06	GC	$C_6H_{10}O_7$	194.0427	0.0356	3627
D-gluconic acid	56.35	2.69	GC	$C_6H_{12}O_7$	196.0583	0.0209	10690

D-glucuronic acid	-41.46	0.02	GC	$C_6H_{10}O_7$	194.0427	0.0039	444791
Ferulic acid	22.34	0.41	FT-	$C_{10}H_{10}O_4$	194.0579	0.0437	445858, 1548883
N-feruloylputrescine	-7.59	3.36	FT+	$C_{14}H_{20}N_2O_3$	264.1473	0.0487	5281796
N-feruloyltyramine	48.08	0.09	FT+	$C_{18}H_{19}NO_4$	313.1314	0.0172	5280537
Nonanedioic acid [d] (Azelaic acid)	41.55	0.08	GC	$C_9H_{16}O_4$	188.1049	0.0009	2266
	24.36	0.02	FT-			0.0451	
Pentanedioic acid (Glutaric acid)	-14.56	0.16	FT-	$C_5H_8O_4$	132.0423	0.0205	743
	-49.39	1.24	GC			<0.0001	
Succinic acid [d]	65.36	1.54	GC	$C_4H_6O_4$	118.0266	<0.0001	1110
Fatty acids and peroxides							
(*Z*)-Octadec-9-enoic acid (Oleate)	-24.61	7.61	FT-	$C_{18}H_{34}O_2$	282.2558	0.0487	445639
	-31.40	0.06	GC			0.0057	
Colneleic acid	84.79	4.26	FT-	$C_{18}H_{30}O_3$	294.2194	0.0012	6441681
Colnelenic acid	20.68	1.51	FT-	$C_{18}H_{28}O_3$	292.2038	0.0457	6441679
Hydroperoxyoctadecatrienoic acid (Hydroperoxylinolenic acid)	-51.54	1.16	FT-	$C_{18}H_{30}O_4$	310.2144	0.0378	5497123, 6440263, 5282866
Hydroxyoctadecadienoic acid (Hydroxylinoleic acid)	26.93	0.09	FT+	$C_{18}H_{32}O_3$	296.2351	0.0346	5282948, 6443013, 5282945, 5312833 5281117, 5312830 6433819, 6440705
	70.96	6.23	FT-				
Octadecadienoic acid (Linolate)	44.34	18.84	FT-	$C_{18}H_{32}O_2$	280.2402	0.0001	5280450
	26.32	0.30	GC			0.0167	
Trihydroxyoctadecanoic acid (Trihydroxystearic acid)	-49.03	1.21	FT-	$C_{18}H_{36}O_5$	332.2563	0.0015	147011
Several groups							
α-Tocotrienol	58.95	2.39	FT-	$C_{29}H_{44}O_2$	424.3341	0.0300	5282347, 92161, 11464420
*7*H-Purin-6-amine (Adenine)	69.82	1.01	GC	$C_5H_5N_5$	135.0545	0.0015	190

	42.88	0.12	FT-			<0.0001	
4-(*1*H-indol-*3*-yl)butanoic acid (Indolebutyric acid)	-62.75	1.16	FT+	$C_{12}H_{13}NO_2$	203.0946	0.0206	8617

[a] Values refer to the relative (%) peak area or amplitude of total peak area or amplitude and represent the mean of eight biological replications

[b] FT+; Fourier transform-ion cyclotron resonance-mass spectrometry (FT-ICR/MS)-positive mode, FT-; FT-ICR/MS-negative mode, GC; gas chromatography MS

[c] not detected in controls

[d] Metabolites that were definitely identified based on matching their mass spectra and retention times to those of the authentic chemical standards analyzed on the same system with the same analytical method.
