# Supplementary material for: FT-ICR/MS and GC-EI/MS Metabolomics Networking Unravels Global Potato Sprout's Responses to Rhizoctonia solani Infection
Source: PLoS One. 2012 Aug 3;7(8):e42576. doi: 10.1371/journal.pone.0042576 (PMC3411821; doi:10.1371/journal.pone.0042576)
Supplement: Table S2 — Identified metabolites of eight-day-old mycelia of Rhizoctonia solani AG3 grown on PDA that could possibly have a high leverage during the biological interpretation of metabolomics data. (PDF) [file pone.0042576.s006.pdf]

Table S2. Identified metabolites of eight-day-old mycelia of *Rhizoctonia solani* AG3 grown on PDA that could possibly have a high leverage during the biological interpretation of metabolomics data. As a rule of thumb, significantly increased metabolites in infected plant tissues as revealed by metabolomics analyses that are also components of fungal mycelia are not biologically interpreted.

Metabolites	Molecular form	Monoisotopic mass (Da)
AMINO ACIDS		
Protein amino acids		
L-alanine[a]	$C_3H_7NO_2$	89.0477
L-proline[a]	$C_5H_9NO_2$	115.0633
L-valine[a]	$C_5H_{11}NO_2$	117.0790
CARBOHYDRATES		
Monosaccharides		
D-glucose[a]	$C_6H_{12}O_6$	180.0634
D-fructose[a]	$C_6H_{12}O_6$	180.0634
N-acetylglucosamine	$C_8H_{15}NO_6$	221.0899
Polysaccharides		
α-*α*-Trehalose[a]	$C_{12}H_{22}O_{11}$	342.1162
Sucrose[a]	$C_{12}H_{22}O_{11}$	342.1162
Maltose	$C_{12}H_{22}O_{11}$	342.1162
Sugar alcohols		
Erythritol	$C_4H_{10}O_4$	122.0579
Glycerol[a]	$C_3H_8O_3$	92.0473
Mannitol	$C_6H_{14}O_6$	182.0790
Myo-inositol[a]	$C_6H_{12}O_6$	180.0634
Tetrose carbohydrates		
Erythrose	$C_4H_8O_4$	120.0423

CARBOXYLIC ACIDS		
Lactic acid	$C_3H_6O_3$	90.0316
Succinic acid[a]	$C_4H_6O_4$	118.0266
FATTY ACIDS		
(*9Z*)-Octadecenoic acid (Oleic acid)	$C_{18}H_{34}O_2$	282.2558
Octadecadienoic acid (Linoleic acid)	$C_{18}H_{32}O_2$	280.2402

[a] Metabolites that were definitely identified based on matching the mass spectra and retention times and the accurate masses of their adducts to those of the authentic chemical standards analyzed on the same system with the same analytical method.
