# Supplementary material for: FT-ICR/MS and GC-EI/MS Metabolomics Networking Unravels Global Potato Sprout's Responses to Rhizoctonia solani Infection
Source: PLoS One. 2012 Aug 3;7(8):e42576. doi: 10.1371/journal.pone.0042576 (PMC3411821; doi:10.1371/journal.pone.0042576)
Supplement: Table S3 — Half maximal effective concentration (EC50) of selected antifungal potato biomarkers against Rhizoctonia solani . (PDF) [file pone.0042576.s007.pdf]

**Table S3.** Half maximal effective concentration ($EC_{50}$) of selected antifungal potato biomarkers against *Rhizoctonia solani*.

| Metabolites | $EC_{50}$ (M) |
|---|---|
| azoxystrobin (commercial fungicide) | 0.007±0.001[a] |
| cinnamate | 0.576±0.151[b] |
| fumarate | 7.156±0.824[c] |
| oxalate | 5.698±1.231[c] |
| $\alpha$-solanine | 0.189±0.052[d] |
| succinate | 5.351±1.051[c] |

[a]Values represent the means of three separate experiments with three replications and standard deviation. Means followed by the same letter are not significantly different (Tukey's HSD test, $P<0.05$).
