# Supplementary material for: FT-ICR/MS and GC-EI/MS Metabolomics Networking Unravels Global Potato Sprout's Responses to Rhizoctonia solani Infection
Source: PLoS One. 2012 Aug 3;7(8):e42576. doi: 10.1371/journal.pone.0042576 (PMC3411821; doi:10.1371/journal.pone.0042576)
Supplement: Table S4 — Potato sprouts' metabolites with the highest fluctuation 72 h after infection by Rhizoctonia solani with enzymes catalyzing their biosynthesis and corresponding encoding genes. (PDF) [file pone.0042576.s008.pdf]

Table S4. Potato sprouts' metabolites with the highest fluctuation 72 h after infection by *Rhizoctonia solani* as revealed performing metabolomics analyses. The connections between those metabolites with enzymes catalyzing their biosynthesis and corresponding encoding genes were based on information retrieved from the databases PotatoCyc and KEGG. In cases where no hits were found in these databases, searches were performed in the PlantCyc database, and results are displayed for other plant species. Linking to the associated on-line sources is provided by clicking on the bold highlighted codes/words holding the shift button.

Metabolite(s)	*Catalyzing enzyme(s)*	*Biosynthetic pathway(s)*	*Accession number*		*Organism*[a]
			PotatoCyc	*PlantCyc*	
β-alanine	glutamate decarboxylase (E.C. 4.1.1.15)	*β*-alanine metabolism [**rn00410**] pantothenate and CoA biosynthesis [**ec00770**]	**SGNU-269741, SGNU-274223, SGNU-277090, SGNU-287538 SGNU-287539, SGNU-287540, SGNU-293317**		*S. tuberosum*
	aspartate 1-decarboxylase (E.C. 4.1.1.11)				
	aldehyde dehydrogenase (NAD+) (E.C. 1.2.1.3)	*β*-alanine metabolism [**rn00410**]	**SGNU-270085**		*S. tuberosum*
	aminobutyraldehyde dehydrogenase (E.C. 1.2.1.19)				
	beta-ureidopropionase (E.C. 3.5.1.6)	pyrimidine metabolism [**ec00240**], *β*-alanine metabolism [**rn00410**], pantothenate and CoA biosynthesis [**ec00770**]		**JGI-819740**<br>**LOC_Os07g30170.1**<br>**PYD3**<br>**TC100937, TC100938, TC108524**	*P. trichocarpa*<br>*O. sativa*<br>*A. thaliana*<br>*M. truncatula*
	β-alanine aminotransferase (E.C. 2.6.1.18)	*β*-alanine metabolism [**rn00410**], propanoate metabolism [**rn00640**]	**AT-3G08860.1**		*A. thaliana*
	N-acetyl-beta-alanine deacetylase (E.C. 3.5.1.21)	*β*-alanine metabolism [**rn00410**]			
	aminoacyl-lysine dipeptidase (E.C. 3.4.13.4)				

	carnosine synthase (E.C. 6.3.2.11)				
	guanidinopropionase (E.C. 3.5.3.17)				
	aminoacylhistidine dipeptidase (E.C. 3.4.13.3)				
	pantothenate amidohydrolase (E.C. 3.5.1.22)	pantothenate and CoA biosynthesis [**rn00770**]			
	anserinase (E.C. 3.4.13.5)	*β*-alanine metabolism [**rn00410**]			
4-aminobutanoic acid (GABA)	glutamate decarboxylase (E.C. 4.1.1.15)	alanine, aspartate and glutamate metabolism [**rn00250**]	**SGN-U269741, SGN-U274223, SGN-U277090, SGNU-287538, SGNU-287539, SGNU-287540 SGNU-293317**		*S. tuberosum*
	aldehyde dehydrogenase (NAD+) (E.C. 1.2.1.3)	arginine and proline metabolism [**rn00330**]	**SGNU-270085**		*S. tuberosum*
	guanidinobutyrase (E.C. 3.5.3.7)				
	aminoacylhistidine dipeptidase (E.C. 3.4.13.3)				
	aminobutyraldehyde dehydrogenase (E.C. 1.2.1.19)<br>feruloyl esterase (E.C. 3.1.1.73)	arginine and proline metabolism [**rn00330**]<br>*β*-alanine metabolism [**rn00410**]			
	gamma-glutamyl-gamma-aminobutyrate hydrolase (E.C. 3.5.1.94)	arginine and proline metabolism [**rn00330**]			
γ-chaconine	UDP-glucose glucotransferase St-SGT2 (E.C. 2.4.1.-)	steroidal glycoalkaloid biosynthesis	**SGNU293662, SGNU283603**		*S. tuberosum*
cinnamic acid	phenylalanine ammonia-lyase (E.C. 4.3.1.24)	phenylalanine metabolism [**rn00360**]<br>nitrogen metabolism [**rn00910**]<br>phenylpropanoid biosynthesis [**ko00940**]		**SGN-U312619, SGN-U312620, SGN-U312621, SGN-U333748, SGN-U333750, SGN-U312617, SGN-U312618, SGN-U325698, SGN-U330655, SGN-U333749, SGN-U333751, SGN-U341447,**	*S. lycopersicum*<br>*A. thaliana*

				**SGN-U345601,**<br>**PAL1, PAL2, PAL3, PAL4**	
	phenylalanine/tyrosine ammonia-lyase;<br>PTAL (E.C. 4.3.1.25)	phenylalanine metabolism [**rn00360**]<br>nitrogen metabolism [**rn00910**]<br>phenylpropanoid biosynthesis [**ko00940**]			
	2-enoate reductase (E.C. 1.3.1.31)	phenylalanine metabolism [**rn00360**]			
	cinnamoyl-CoA:phenyllactate CoA-<br>transferase (E.C. 2.8.3.17) FldA	tropane, piperidine and pyridine alkaloid<br>biosynthesis [**rn00960**]			
colneleic acid	9-divinyl ether synthase (St), St-StDES	**divinyl ether biosynthesis I (9-LOX)**		G-9759	*S. tuberosum*
colnelenic acid	9-divinyl ether synthase (St), St-StDES	**divinyl ether biosynthesis I (9-LOX)**		G-9759	*S. tuberosum*
ferulic acid	4-coumarate--CoA ligase (E.C. 6.2.1.12)	phenylpropanoid biosynthesis [**rn00940**]	**SGNU269219, SGNU269220**<br>**SGNU269221, SGNU269458,**<br>**SGNU269459, SGNU271690,**<br>**SGNU273689, SGNU276387,**<br>**SGNU279444, SGNU281563,**<br>**SGNU281995, SGNU287264,**<br>**SGNU287264, SGNU287265,**<br>**SGNU287266, SGNU292841,**<br>**SGNU293253, SGNU294251**<br>**SGNU296205, SGNU297674**		*S. tuberosum*
	caffeic acid 3-O-methyltransferase<br>(E.C. 2.1.1.68)	phenylpropanoid biosynthesis [**ko00940**]		**1481.m00053, BE319602**<br>**BG646036, BI311272,**<br>**OMT1, TC100776,**<br>**TC100776, TC102340,**<br>**TC98964**	*M. truncatula*
	feruloyl esterase (E.C. 3.1.1.73)	phenylpropanoid biosynthesis [**ko00940**]			
	deacylase (E.C. 3.5.1.71)	phenylpropanoid biosynthesis [**ko00940**]			
	caffeic acid 3-O-methyltransferase<br>(E.C. 2.1.1.68)	phenylpropanoid biosynthesis<br>[**rn00940**]/**Suberin biosynthesis**		**1481.m00053**<br>**BE319602**<br>**BG646036**<br>**BI311272**	*M. truncatula*

				**TC100776**<br>**TC102340**<br>**TC98964**<br>**OMT1**	*A. thaliana*
	N-feruloylglycine deacylase (E.C. 3.5.1.71)				
	coniferyl-aldehyde dehydrogenase (E.C. 1.2.1.68)	phenylpropanoid biosynthesis [**rn00940**]			
	trans-feruloyl-CoA synthase (E.C. 6.2.1.34)				
	feruloyl esterase (E.C. 3.1.1.73)				
	ferulate-5-hydroxylase (E.C. 1.14.-.-)				
	sinapate 1-glucosyltransferase (E.C. 2.4.1.120)			**AT3G21560, AT4G15480, AT4G15490, AT4G15500, SGT1**	*A. thaliana*<br>*B. napus*<br>*R. sativus*
	hydroxycinnamate 4-beta-glucosyltransferase (E.C. 2.4.1.126)				
feruroyltyramine	tyramine N-feruloyltransferase (E.C. 2.3.1.110)	suberin biosynthesis	**SGNU269846, SGNU269847, SGNU283523, SGNU287587, SGNU287588**		*S. tuberosum*
fumaric acid	**fumarate hydratase** (E.C. 4.2.1.2)	phenylalanine degradation I Citrate cycle (TCA cycle) [**ko00020**]	**SGNU273248**		*S. tuberosum*
	**fumarylacetoacetase** (E.C. 3.7.1.2)	phenylalanine degradation I	**SGNU290011, SGNU290010, SGNU290009, SGNU278057, SGNU271822**		*S. tuberosum*
	**succinate dehydrogenase** (E.C. 1.3.99.1)	citrate cycle (TCA cycle) [**ko00020**], butanoate metabolism [**rn00650**], reductive carboxylate cycle (CO_2 fixation) [**rn00720**], benzoate degradation via CoA ligation [**rn00632**]			

	fumarate reductase (NADH) (E.C. 1.3.1.6)				
	aspartate ammonia-lyase (E.C. 4.3.1.1)	alanine, aspartate and glutamate metabolism [**rn00250**], nitrogen metabolism [**rn00910**]	**SGNU269166, SGNU274897**		*S. tuberosum*
	glutamin-(asparagin-)ase (E.C. 3.5.1.38.)				
	adenylosuccinate lyase (E.C. 4.3.2.2)	purine metabolism [rn00230] alanine, aspartate and glutamate metabolism [**rn00250**]	**SGNU284950, SGNU290676**		*S. tuberosum*
	acylpyruvate hydrolase (E.C. 3.7.1.5)	tyrosine metabolism [**rn00350**]			
	argininosuccinate lyase (E.C. 4.3.2.1)	alanine, aspartate and glutamate metabolism [**rn00250**], arginine and proline metabolism [**rn00330**]	**SGNU280046, SGNU280873**		*S. tuberosum*
	maleate isomerase (E.C. 5.2.1.1)	butanoate metabolism [**rn00650**], nicotinate and nicotinamide metabolism [**rn00760**]			
	carboxymethyloxysuccinate lyase (E.C. 4.2.99.12)				
	fumarylacetoacetase (E.C. 3.7.1.2)	tyrosine metabolism [**rn00350**], styrene degradation [**rn00643**]	**SGNU271822, SGNU278057, SGNU290009, SGNU290010, SGNU290011**		*S. tuberosum*
fumaric acid	succinate dehydrogenase (E.C. 1.3.5.1)	citrate cycle (TCA cycle) [**rn00020**]		**AT2G18450, AT3G27380, AT5G65165**	*A. thaliana*
				**BQ138479**	*M. truncatula*
				**JGI-219180, JGI-593943**	*P. trichocarpa*
				**LOC_Os07g04240.1, LOC_Os09g20440.1**	*O. sativa*
	adenylosuccinate lyase (E.C. 4.3.2.2)	purine metabolism [**rn00230**]	**SGNU284950, SGNU290676**		*S. tuberosum*
	benzylsuccinate synthase (E.C. 4.1.99.11)	benzoate degradation via CoA ligation [**rn00632**]			
	2-hydroxy-6-ketonona-2,4-dienedioic acid hydrolase (E.C. 3.7.1.-)	phenylalanine metabolism [**rn00360**]			
	lyases (E.C. 4.1.99.-)	1- and 2-Methylnaphthalene degradation			

		[**rn00624**]			
D-galactaric acid	uronate dehydrogenase (E.C. 1.1.1.203)<br>galactarate dehydratase (E.C. 4.2.1.42)	ascorbate and aldarate metabolism<br>[**ko00053**]			
gluconic acid	gluconolactonase (E.C. 3.1.1.17)	pentose phosphate pathway<br>[**ko00030**]			
	gluconate 2-dehydrogenase<br>(E.C. 1.1.1.215)				
glyceric acid	glycerate dehydrogenase<br>(E.C. 1.1.1.29)	glycine, serine and threonine metabolism<br>[**rn00260**]<br>glyoxylate and dicarboxylate metabolism<br>[**rn00630**]	**SGNU272307, SGNU288419**		*S. tuberosum*
	hydroxypyruvate reductase<br>(E.C. 1.1.1.81)		**SGNU283355, SGNU286066**		*S. tuberosum*
	gyoxylate/hydroxypyruvate reductase A<br>(E.C. 1.1.1.79)				
	glyoxylate reductase (E.C. 1.1.1.26				
	glyoxylate reductase (NADP+)<br>(E.C. 1.1.1.79)				
	3-phosphoglycerate phosphatase<br>(E.C. 3.1.3.38)	glycine, serine and threonine metabolism<br>[**rn00260**]			
	glycerate kinase (E.C. 2.7.1.31)	glycine, serine and threonine metabolism<br>[**rn00260**], glycerolipid metabolism<br>[**rn00561**], glyoxylate and dicarboxylate<br>metabolism [**rn00630**]		**AT1G80380**<br>**JGI-415047, JGI-829520**	*A. thaliana*<br>*P. trichocarpa*
	2-hydroxy-3-oxopropionate reductase (E.C.<br>1.1.1.60)	glyoxylate and dicarboxylate metabolism<br>[**rn00630**]	**SGNU278460, SGNU285986**		*S. tuberosum*
	phosphoglycerate phosphatase<br>(E.C. 3.1.3.20)				
	oxaloglycolate reductase (decarboxylating)<br>(E.C. 1.1.1.92)	glyoxylate and dicarboxylate metabolism<br>[**rn00630**]			
	tartrate decarboxylase (E.C. 4.1.1.73)				

	aldehyde dehydrogenase (NAD+) (E.C. 1.2.1.3)	glycerolipid metabolism [**rn00561**]	**SGNU270085**	*S. tuberosum*
	kinases (E.C. 2.7.1.-)		**SGNU271185, SGNU274832, SGNU276713, SGNU276826, SGNU277635, SGNU277961, SGNU281005, SGNU281971, SGNU282732, SGNU284158, SGNU286178, SGNU288877, SGNU292150, SGNU295240**	*S. tuberosum*
linoleic acid	lipoxygenase (E.C. 1.13.11.12)	linoleic acid metabolism [**rn00591**]	**SGNU268108, SGNU268109, SGNU268110, SGNU269397, SGNU275813, SGNU283623, SGNU285664, SGNU286657, SGNU286658, SGNU286659, SGNU291453, SGNU294974, SGNU296125**	*S. tuberosum*
	arachidonate 15-lipoxygenase (E.C. 1.13.11.33)			
	linoleate isomerase (E.C. 5.2.1.5)			
	linoleate 11-lipoxygenase (E.C. 1.13.11.45)			
	cytochrome P450 1.14.14.1)		**SGNU276200, SGNU291486**	*S. tuberosum*
	arachidonate 5-lipoxygenase (E.C. 1.13.11.34)			
	linoleate (8R)-dioxygenase (E.C. 1.13.11.44)			
	linoleoyl-CoA desaturase (E.C. 1.14.19.3)			
	phospholipase A (E.C. 3.1.1.4)		**SGNU278771, GNU278772, SGNU278773, SGNU278774, SGNU278776, SGNU278777,**	*S. tuberosum*

			**SGNU278781, SGNU283241, SGNU283241, SGNU289839, SGNU289846**		
	palmitoyl-CoA hydrolase (E.C. 3.1.2.2)			**JGI-663966, JGI-675790**	*P. trichocarpa*
oxalic acid	**oxalate oxidase (**E.C. 1.2.3.4)	glyoxylate and dicarboxylate metabolism [**ko00630**]			
	oxaloacetase (E.C. 3.7.1.1)				
	glyoxylate oxidase (E.C. 1.2.3.5)				
	oxalate decarboxylase (E.C. 4.1.1.2)				
	ascorbate 2,3-dioxygenase (E.C. 1.13.11.13)				
	oxalyl coenzyme A synthetase (E.C. 6.2.1.8)				
	oxalate coenzyme A-transferase (E.C. 2.8.3.2)				
	formyl-CoA transferase (E.C. 2.8.3.16)				
pipecolic acid	saccharopine dehydrogenase (NAD+, L-glutamate forming) (E.C. 1.5.1.9)	tropane, piperidine and pyridine alkaloid biosynthesis [**rn00960**]		**JGI-739274, JGI-739281**	*P. trichocarpa*
	saccharopine dehydrogenase (NAD+, L-lysine forming) (E.C. 1.5.1.7)				
	lysine 6-dehydrogenase (E.C. 1.4.1.18)				
pyroglutamic acid	aminoacyltransferase (E.C. 2.3.2.-)	glutathione metabolism [**rn00480**], butirosin and neomycin biosynthesis [**rn00524**]	**SGNU278180, SGNU279602, SGNU293093**		*S. tuberosum*
sesquiterpenoid phytoalexins	vetispiradiene synthase, VS1 (E.C. 4.2.3.21)	biosynthesis of alkaloids derived from terpenoid and polyketide [**map01066**]/**sesquiterpenoid phytoalexins biosynthesis**		**VS1**	*H. muticus*
sesquiterpenoid phytoalexins & steroidal glycoalkaloids	3-hydroxy-3-methylglutaryl-CoA reductase, HMG1 (E.C. 1.1.1.34)	terpenoid backbone biosynthesis [**map00900**], biosynthesis of alkaloids derived from terpenoid and polyketide [**map01066**]		**AW687494, BE124699, BG644380, BQ140041, CB892390, TC102857, TC106632**	*M. truncatula*

				**HMG1**	*A. thaliana*
				**JGI-595031, JGI-798252, JGI-820962, JGI-829982, JGI-831722, JGI-834149**	*P. trichocarpa*
				**LOC_Os01g31610.1, LOC_Os01g34270.1, LOC_Os02g48330.1, LOC_Os03g38870.1, LOC_Os05g02990.1, LOC_Os05g38960.1, LOC_Os08g40180.1, LOC_Os09g31970.1**	*O. sativa*
sesquiterpenoid phytoalexins & steroidal glycoalkaloids	1-deoxy-D-xylulose-5-phosphate reductoisomerase, dxr (E.C. 1.1.1.267)	terpenoid backbone biosynthesis [**ko00900**], biosynthesis of alkaloids derived from terpenoid and polyketide [**map01066**]	**SGNU270961, SGNU270962, SGNU270963**		*S. tuberosum*
sesquiterpenoid phytoalexins & steroidal glycoalkaloids	farnesyl diphosphate synthase, ispA (E.C. 2.5.1.1 2.5.1.10)	terpenoid backbone biosynthesis [**ko00900**], biosynthesis of alkaloids derived from terpenoid and polyketide [**map01066**]	**SGNU269148, SGNU269149, SGNU287228, SGNU287229**		*S. tuberosum*
γ-solanine	UDP-galactose galactosyltransferase St-SGT1 (E.C. 2.4.1.-)	biosynthesis of alkaloids derived from terpenoid and polyketide [**map01066**]	**SGNU269176, SGNU270492, SGNU270493, SGNU275270, SGNU275759, SGNU277244, SGNU278383, SGNU279601, SGNU280444, SGNU283603, SGNU287245, SGNU289954, SGNU290507, SGNU293303 SGNU293662, SGNU298857**		*S. tuberosum*
steroidal glycoalkaloids	farnesyl-diphosphate farnesyltransferase, FDFT1 (E.C. 2.5.1.21)	steroid biosynthesis [**ko00100**], biosynthesis of alkaloids derived from terpenoid and polyketide [**map01066**]	**SGNU270583, SGNU287853**		*S. tuberosum*

steroidal glycoalkaloids	squalene monooxygenase, SQLE (E.C. 1.14.99.7)	steroid biosynthesis [**ko00100**], biosynthesis of alkaloids derived from terpenoid and polyketide [**map01066**]	**SGNU291972, SGNU269909**		*S. tuberosum*
succinic acid	succinyl-CoA synthetase alpha subunit, LSC1 (E.C. 6.2.1.5)	citrate cycle (TCA cycle) [**ko00020**], propanoate metabolism [**ko00640**], C5-branched dibasic acid metabolism [ko00660], reductive carboxylate cycle (CO_2 fixation) [**ko00720**]	**SGNU268134, SGNU270065, SGNU287678, SGNU287677, SGNU270066**		*S. tuberosum*
	succinate dehydrogenase (ubiquinone) flavoprotein subunit (E.C. 1.3.5.1)	citrate cycle (TCA cycle) [**ko00020**], oxidative phosphorylation [**ko00190**]		**AT2G18450, AT3G27380, AT5G65165**	*A. thaliana*
				**BQ138479**	*M. truncatula*
				**JGI-219180, JGI-593943**	*P. trichocarpa*
				**LOC_Os07g04240.1**	*O. sativa*
				**LOC_Os09g20440.1**	
	succinate dehydrogenase flavoprotein subunit, sdhA (E.C. 1.3.99.1)	citrate cycle (TCA cycle) [**ko00020**], oxidative phosphorylation [**ko00190**] benzoate degradation via CoA ligation [ko00632], butanoate metabolism [**ko00650**], reductive carboxylate cycle (CO2 fixation) [**ko00720**]			
	succinyl-CoA synthetase alpha subunit, LSC1 (E.C. 6.2.1.4 6.2.1.5)	citrate cycle (TCA cycle) [**ko00020**], propanoate metabolism [**ko00640**]	**SGNU283178, SGNU-268134, SGNU270065, SGNU287678, SGNU-287677, SGNU-270066**		*S. tuberosum*

[a] *A. thaliana; Arabidopsis thaliana* (Arabidopsis), *B. napus*; *Brassica napus* (rapeseed), *H. muticus*; *Hyoscyamus muticus* (henbane), *M. truncatula; Medicago truncatula* (barrel clover), *O. sativa; Oryza sativa* (rice), *P. trichocarpa; Populus trichocarpa* (black cottonwood), *R. sativus*; *Raphanus sativus* (radish), *S. lycopersicum*; *Solanum lycopersicum* (tomato), *S. tuberosum; Solanum tuberosum* (potato).
